# A Homeostatic Sleep-Stabilizing Pathway in *Drosophila* Composed of the Sex Peptide Receptor and Its Ligand, the Myoinhibitory Peptide

**DOI:** 10.1371/journal.pbio.1001974

**Published:** 2014-10-21

**Authors:** Yangkyun Oh, Sung-Eun Yoon, Qi Zhang, Hyo-Seok Chae, Ivana Daubnerová, Orie T. Shafer, Joonho Choe, Young-Joon Kim

**Affiliations:** 1 Department of Biological Sciences, Korea Advanced Institute of Science and Technology, Daejeon, South Korea; 2 School of Life Sciences, Gwangju Institute of Science and Technology, Gwangju, South Korea; 3 Department of Molecular, Cellular, and Developmental Biology, University of Michigan, Ann Arbor, Michigan, United States of America; Washington University, United States of America

## Abstract

Sleep, a reversible quiescent state found in both invertebrate and vertebrate animals, disconnects animals from their environment and is highly regulated for coordination with wakeful activities, such as reproduction. The fruit fly, *Drosophila melanogaster*, has proven to be a valuable model for studying the regulation of sleep by circadian clock and homeostatic mechanisms. Here, we demonstrate that the sex peptide receptor (SPR) of *Drosophila*, known for its role in female reproduction, is also important in stabilizing sleep in both males and females. Mutants lacking either the SPR or its central ligand, myoinhibitory peptide (MIP), fall asleep normally, but have difficulty in maintaining a sleep-like state. Our analyses have mapped the SPR sleep function to *pigment dispersing factor* (*pdf*) neurons, an arousal center in the insect brain. MIP downregulates intracellular cAMP levels in *pdf* neurons through the SPR. MIP is released centrally before and during night-time sleep, when the sleep drive is elevated. Sleep deprivation during the night facilitates MIP secretion from specific brain neurons innervating *pdf* neurons. Moreover, flies lacking either SPR or MIP cannot recover sleep after the night-time sleep deprivation. These results delineate a central neuropeptide circuit that stabilizes the sleep state by feeding a slow-acting inhibitory input into the arousal system and plays an important role in sleep homeostasis.

## Abstract 2

A ligand of the sex peptide receptor maintains sleep stability and homeostasis by inhibiting wakefulness-promoting neurons in *Drosophila*.

## Author Summary

Sleep is a common trait in animals, from insects to mammals, and it needs to be coordinated with other critical activities such as feeding and reproduction. However, the mechanisms by which this is achieved are not fully understood. The fruit fly *Drosophila melanogaster* has become a key model organism for sleep research and it has been shown that reproduction is one of the factors that can modulate sleep in these animals. Researchers have observed that mating reduces the daytime sleep of female flies and shown that the seminal fluid protein Sex Peptide (SP), a ligand of the Sex Peptide Receptor (SPR) that is transferred to females during copulation, is responsible for this reduction of siesta sleep. Here, we investigated further the role of SPR in sleep regulation in *Drosophila*. We show that SPR is required for sleep stabilization in both sexes and that in mutant flies lacking SPR or its ligand myoinhibitory peptide (MIP) sleep is fragmented independently of reproduction. Unlike SP, MIP is expressed in the brain of both sexes and acts on SPR to silence specific neurons that keep flies awake, stabilizing sleep. Hence, our results reveal that SPR interacts with two distinct ligands to control different behaviors: SP for reproduction and MIP for sleep.

## Introduction

Sleep is an evolutionarily conserved physiological state marked by sustained and reversible quiescence during which animals display reduced responsiveness to external stimuli [1]. Sleep is important for diverse biological processes, such as immune responses, metabolism, obesity, longevity, and learning and memory, and reduction in the quality and quantity of sleep in humans, can give rise to sleep disorders and increased morbidity [2],[3]. Despite its medical importance and wide occurrence across animal phylogeny, the evolutionary and functional origins of sleep remain poorly understood [4].

Recently, the fruit fly *Drosophila melanogaster* has become an important invertebrate model for sleep research [5],[6]. This genetically amenable organism has a simpler central nervous system (CNS) and shares defining characteristics of mammalian sleep, such as reduced sensory responsiveness, dual circadian and homeostatic regulation, and reduced brain activity [7]–[10]. Genetic studies on *Drosophila* have identified many molecules and pathways important for sleep control, many of which have conserved roles in regulating mammalian sleep [1],[5]. As in mammals, GABAergic signaling promotes sleep in *Drosophila* mainly by suppressing activities of the arousal systems [4],[11],[12]. Studies on a GABA_A_ receptor mutant revealed that sleep onset and sleep maintenance are genetically dissociable [2],[3],[13]. However, how sleep initiation and maintenance are differentially regulated is not known.

Sleep is governed mainly by two regulatory systems: circadian and homeostatic drive. The molecular model that explains circadian control of the wake-sleep cycle is well established (for a review, [5]). In contrast, no coherent mechanism for sleep homeostasis is yet available. Sleep is also shaped by other competing or complementary behaviors, such as ones associated with learning, feeding, and reproduction [5],[6],[14]. Mating was shown to induce female *Drosophila* to sleep less, particularly during the daytime [15]. Sex peptide (SP), a male seminal protein transferred to the female during copulation [16],[17], was implicated in the post-mating reduction of siesta sleep, because females that copulated with males lacking *SP* did not lose sleep. Sex peptide receptor (SPR), a G-protein coupled receptor (GPCR) mediates many of the actions of SP on female reproductive behavior [18]. On transfer to the female, SP activates SPR in a small number of uterine neurons and triggers post-mating behavioral responses (PMR), characterized by (but not restricted to) suppression of mating receptivity and initiation of egg laying [19],[20].

For the PMR induction, SPR is required only in a small number of female-specific internal sensory neurons. However, SPR expression in the CNS is broad, and shows little sexual difference, suggesting it has additional roles in both males and females [18]. Recently, the brain-gut myoinhibitory peptides (MIPs) (also known as allatostatin-B or prothoracicostatic peptides), have been shown to also activate SPR when expressed heterologously in mammalian cells [21]–[23]. Unlike SP, however, MIP, which is expressed in the central interneurons with little sexual dimorphism [24], is unable to induce the PMR in *Drosophila* females [21]. Thus, the biological function of MIP-SPR signaling in *Drosophila* remains elusive.

Here, we demonstrate that mutants lacking either SPR or its ligand, MIP, slept less regardless of sex and mating status, primarily because of difficulty in maintaining sleep. By combining genetic analyses and optical activity imaging, we found that SPR mediates MIP actions to modulate neural activities of a fly arousal system involving *pigment dispersing factor* (*pdf*) neurons. The brain MIP neurons release MIP before and during night-time sleep. Sleep deprivation facilitates MIP secretion from a small subset of brain neurons, axonal processes of which innervate dendritic fields of *pdf* neurons. Mutants lacking either SPR or MIP failed to show normal sleep homeostasis. We conclude that the MIP-SPR signaling pathway functions as a sleep homeostat that senses the need for sleep and stabilizes sleep by providing a slow-acting inhibitory input to an arousal center.

## Results

### *SPR* Is Important for Sleep Maintenance

**Figure 1:**
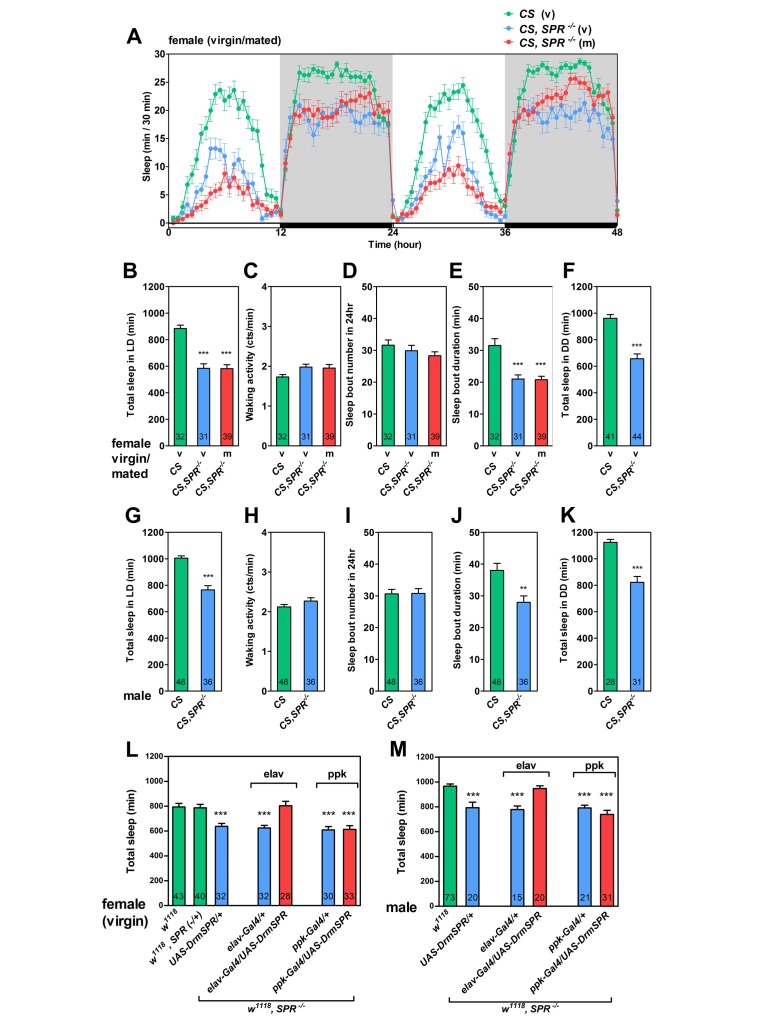
SPR expression in the CNS is essential for sleep maintenance. (A) Standard sleep plots of *CS* control and cantonized *SPR*-deficient mutant females (*CS,SPR* ^−/−^) under 12-h∶12-h light∶dark (L∶D). Shaded boxes depict dark periods. (v) and (m) indicate virgin and mated females, respectively. (B–F, L) Sleep parameter of females of indicated genotypes. Females used in (L) are virgin. (G–K, M) Sleep parameter of males of indicated genotypes. (B, G, L, M) Total sleep duration per day. (C, H) Waking activity. (D, I) Sleep bout number per day. (E, J) Mean sleep-bout duration. (F, K) Total sleep duration per day under 12-h∶12-h D∶D condition. Flies used in these assays formed a separate cohort to those in (A–E) and (G–J) (see Figure S2 for complete data). Number in bars indicates *n* of the tested flies. Data are shown as means ± standard error of the mean (SEM). **, *p*<0.01; ***, *p*<0.001 for the comparison to *CS* or *w^1118^* by Student's *t* test (B–D, F–I, K–M) and Mann-Whitney U test (E, J).

To determine whether SPR was necessary for the SP-induced daytime sleep loss, we examined the sleep profile of the *SPR*-deficient mutants (*SPR^−/−^*) that carry a micro-deletion of the genomic region containing *SPR* and a few neighboring genes. The *SPR^−/−^* females fail to switch reproductive behaviors upon mating or SP injection [18]. To control for possible genetic background differences, we compared wild-type *Canton-S* (*CS*) flies with *SPR*-deficient mutants backcrossed with *CS* for six generations. We examined the sleep profiles of virgin females, mated females and males separately. Strikingly, the *SPR*-deficient mutants slept less than the isogenic wild-type counterparts regardless of sex and mating status (virgin versus mated) (Figure 1A–1K). However, virgin and mated females demonstrated almost equal levels of daily sleep in any given genotype (Figures 1B and S1). In *CS* females, the mating effects on siesta sleep are temporary and do not last more than three days after mating (RE Isaac, unpublished data). Because we examined the sleep profile of females at least 5 days after mating, this observation may explain the absence of a mating effect on sleep in our experiments. Nevertheless, lack of SPR expression suppressed both day- and night-time sleep duration by approximately 48% and 77%, respectively, compared to *CS* virgin females (Figure 1A and 1B). This observation contrasts with the daytime sleep loss reported previously for mated females, suggesting that SPR has a role in sleep regulation independent of mating status.

In terms of waking activity, however, *SPR*-deficient mutants did not differ from *CS*, indicating that the sleep loss observed in mutants is not attributable to nonspecific hyperactivity (Figure 1C and 1H). We also measured the number of sleep bouts and the average length of each sleep bout, which indicates sleep initiation and sleep maintenance abilities, respectively. The *SPR*-deficient mutant had almost the same number of sleep bouts, but markedly shorter average sleep-bout length compared to wild-type flies (Figure 1D, 1E, 1I, and 1J). Since environmental light was shown to affect sleep in *Drosophila* [25], we examined the baseline sleep of the SPR deficient mutant in light-dark (LD) and dark-dark (DD) environments. In both conditions, the SPR mutant displayed significant reductions in daytime and night-time sleep (Figures 1F, 1K, S2A, and S2B).

To test whether SPR expression in the nervous system is sufficient for normal sleep behavior, we introduced *elav-Gal4* and upstream activation sequence (*UAS*)-*Drosophila SPR* (*DrmSPR*) into the *SPR*-deficient mutant lines. In these flies, we observed complete rescue of the sleep phenotype in both sexes (Figure 1L and 1M). Subsequently, we tested whether the sleep phenotype was rescued by expressing SPR in *pickpocket* (*ppk*) neurons, in which SPR expression is essential for the SP-induced PMR [19],[20]. Unlike PMR, sleep was not restored by SPR expression in *ppk* neurons, indicating that the sleep-regulating SPR circuit can be separated from the PMR-regulating SPR circuit (Figure 1L). On the basis of these results, we conclude that SPR is essential for baseline sleep maintenance in both males and females.

### Identification of the Sleep-Regulating SPR Circuit

**Figure 2:**
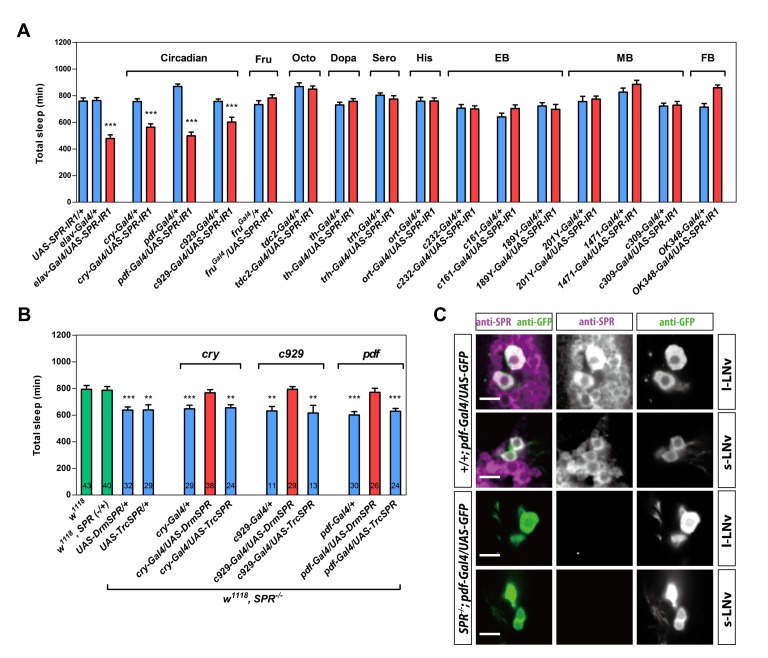
The sleep function of SPR is mapped to *pdf* neurons. (A–B) Total sleep duration per day of virgin females of the indicated genotypes. Data are shown as means ± SEM. In (A), *n* = 16–64 for each bar. ***, *p*<0.001 for the comparison both *Gal4* and *UAS* controls by Student's *t* test. In (B), number in bars indicates *n* of the tested flies. **, *p*<0.01, ***, *p*<0.001 for the comparison to *w^1118^* controls by Student's *t* test. (C) *pdf* neurons express SPR. Confocal sections of the female brain of indicated genotypes stained by anti-SPR (magenta) and anti-GFP (green). Magenta and green channels are shown separately. Each brain hemisphere has four to five l-LNvs and four s-LNvs, most of which are labeled by anti-SPR (*n* = 6). Note that SPR expression is broad and not restricted to *pdf* neurons. Scale bar, 10 µm.

To map the SPR neurons responsible for sleep regulation, we suppressed SPR expression in the major sleep circuits using *SPR-RNAi*. First, we confirmed *SPR-RNAi* efficacy on the sleep phenotype by examining pan-neural *SPR-RNAi (elav-Gal4/UAS-SPR-IR1)* (Figure S3). Like *SPR*-deficient mutants, pan-neural *SPR-RNAi* flies slept considerably less than controls, regardless of sex and mating status, and their total sleep loss was attributable mainly to reduced sleep-bout duration (Figure S3). Next, we ran the *SPR-RNAi* screen with 15 *Gal4* drivers, targeting either the sex-behavior circuit or known sleep-control circuits, including ellipsoid body, mushroom body, fan-shaped body, monoaminergic, and circadian clock neurons (Figure 2A). Knockdown of SPR expression in *fruitless^Gal4^* neurons [26],[27] did not produce the sleep phenotype, but it did result in defects in the female PMR [18]. Again, this result dissociates the SPR circuit regulating sleep from the circuit regulating sexual behavior.

In this screen, we identified three circadian clock-specific *Gal4* neural populations in which SPR expression is required for wild-type levels of sleep (Figure 2A): *cry-Gal4* [28], *C929-Gal4* [29], and *pdf-Gal4* [30]. SPR knockdown resulted in significant sleep loss with all three *Gal4* lines. To test whether SPR expression in these *Gal4* neurons was sufficient for wild-type sleep levels, we combined each of these *Gal4* drivers with *UAS-DrmSPR* in *SPR*-deficient mutants. When SPR expression was restored in *cry-Gal4*, *C929-Gal4*, or *pdf-Gal4* neurons, the sleep phenotype was completely rescued (Figure 2B). However, expression of *Tribolium castaneum* SPR (TrcSPR), which is insensitive to both SP and MIP (see Materials and Methods), did not rescue. Overexpression of DrmSPR or *Aedes aegypti* SPR (AeaSPR) in control background using the pan-neural *elav-Gal4* did not elevate sleep levels (Figure S4).

### SPR Is Expressed in Both Large and Small Lateral Ventral Neurons

Among three *Gal4* neuron populations in which SPR expression was essential for sleep function, *pdf-Gal4* had the most restricted expression pattern. Thus, we investigated whether *pdf* neurons express SPR by staining brains in which *pdf* neurons produce enhanced green fluorescent protein (EGFP) with anti-SPR antibody. The *pdf-Gal4* neurons are largely divided into two groups: the large and small lateral ventral neurons (l-LNvs and s-LNvs). We detected anti-SPR staining in both groups of *pdf* neurons, as well as many other CNS cells (Figure 2C). As expected, *pdf* neurons in brains of SPR-deficient mutants did not stain with the SPR antibody (Figure 2C).

Previously, Rosbash and colleagues used microarray technology to identify 1,000 to 2,000 mRNAs enriched in either l-LNvs or s-LNvs as compared to the population of neurons labeled by the pan-neuronal driver *elav-Gal4* [31]. We were able to verify from their results that SPR is significantly enriched in l-LNvs, but not in s-LNvs. It should be noted that lack of enrichment does not mean absence of expression.

Recently, it was reported that s-LNvs release the inhibitory short neuropeptide F (sNPF), which stabilizes night-time sleep by acting on l-LNvs [32]. Since this result implicated differential roles of l-LNvs and s-LNvs in sleep regulation, we examined day- and night-time sleep separately in *SPR-RNAi* targeting either *pdf-Gal4* neurons, including both l-LNvs and s-LNvs, or *C929-Gal4*, including l-LNvs and other peptidergic secretory neurons, but not s-LNvs. Knockdown of SPR expression in l-LNvs with *C929-Gal4* reduced night-time sleep, but not daytime sleep (Figure S5A–S5C), and sleep-bout duration was only reduced during night-time (Figure S5D and S5E). In addition, knockdown of SPR in both l- and s-LNvs with *pdf-Gal4* reduced day- and night-time sleep and average sleep-bout duration together (Figure S5F–S5J), suggesting that SPR in s-LNvs regulates sleep in daytime. Together, these results suggest that SPR modulates functions of s-LNvs and l-LNvs, resulting in stabilization of daytime and night-time sleep, respectively.

### MIP Is Important for Sleep Maintenance

**Figure 3:**
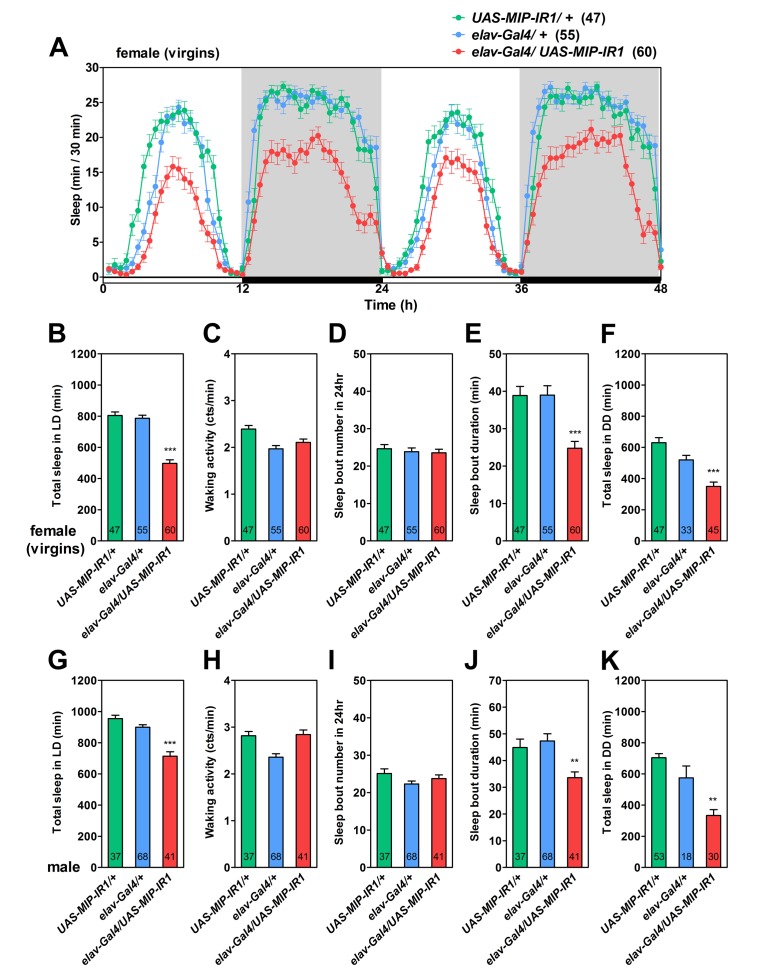
*MIP* encoding SPR ligands is required to stabilize sleep. (A) Standard sleep plots of pan-neural *MIP-RNAi* (*elav-Gal4, UAS-MIP-IR1*) and its control virgin females in a 12-h∶12-h light∶dark cycle (L∶D). Shaded boxes depict dark periods. (B–K) Sleep parameter of virgin females (B–F) and males (G–K) of indicated genotypes. (B, G) Total sleep duration per day. (C, H) Waking activity. (D, I) Sleep bout number per day. (E, J) Mean sleep-bout duration. (F, K) Total sleep duration per day under 12-h∶12-h D∶D condition. Flies used in these assays formed a separate cohort to those in (A–E) and (G–J) (see Figure S2 for complete data). Number in bars indicates *n* of the tested flies. Data are shown as means ± SEM. **, *p*<0.01; ***, *p*<0.001 for the comparison to both *Gal4* and *UAS* controls by Student's *t* test (all except E, J) and Mann-Whitney U test (E, J).

SPR has two unrelated peptide ligands, SP and MIP. Unlike SP, MIP is expressed in central neurons of both males and females [21],[33]. Because we observed a sleep phenotype in both sexes of *SPR*-deficient mutants, we suspected that MIP rather than SP is the sleep-regulating ligand of SPR. To test this hypothesis, we examined the sleep profile upon knockdown of *MIP* expression in the nervous system (*elav-Gal4, UAS-MIP-IR*; henceforth referred to as *MIP-RNAi*). To control for potential off-target effects, two independent RNAi lines (*UAS-MIP-IR1* and *UAS-MIP-IR2*) were tested in parallel (Figures 3 and S6). Anti-MIP staining in the CNS confirmed the knockdown of MIP in two *RNAi* lines (Figure S7). Both sexes of *MIP-RNAi* flies slept less than controls. Sleep loss was evident both day and night and was due to shortened sleep-bout duration, but not changes in the number of sleep bouts. Moreover, like the SPR deficient mutant, *MIP-RNAi* flies sleep less in both LD and DD conditions (Figures 3, S2C, and S2D). These sleep phenotypes are strikingly similar to those observed in *SPR-RNAi* and *SPR*-deficient mutants, further indicating that MIPs are the sleep-regulating ligands for SPR.

Some insect SPRs are highly sensitive to *Drosophila* MIP, but much less so to *Drosophila* SP [18],[21]–[23]. For example, DrmSPR is highly sensitive to MIP (EC_50_, 0.5 nM) and SP (EC_50_, 4.3 nM), whereas AeaSPR is sensitive to MIP (EC_50_, 0.5 nM) but less so to SP (EC_50_, 167 nM). Likewise, *Bombyx mori* SPR (BomSPR) is highly sensitive to MIP (EC_50_, 7.6 nM), but shows modest sensitivity to SP (EC_50_, 67 nM). Hence, we asked whether DrmSPR that is sensitive to both MIP and SP could be functionally substituted by AeaSPR and BomSPR, both of which are sensitive to MIP but not to SP. Pan-neural expression of these SPRs rescued the sleep phenotype of *SPR*-deficient mutants completely, but expression of TrcSPR, which is insensitive to both SP and MIP, did not (Figure S8). Together, these results provide additional support for MIP as both a pharmacologically and behaviorally relevant ligand for the SPR.

### Adult-Specific Knockdowns of SPR or MIP Reduce Sleep

To control indirect developmental effects of SPR or MIP knockdown on sleep, we adopted the RU486-activated gene switch (GS)-Gal4 system [34] and tested whether adult-restricted knockdown of SPR or MIP also produces the short sleep phenotype. *SPR-RNAi* adults carrying *pdf-GS-Gal4* were fed with RU486 or vehicle-containing food for two days prior to the sleep measurement (Figure S9A). Compared with vehicle-treated controls, RU486-treated adults showed significant reductions in daytime and night-time sleep (Figure S9B and S9C), underscoring the adult-specific function of SPR in baseline sleep regulation. In parallel experiments, we examined *MIP-RNAi* combined with *elav-GS-Gal4*. Like *SPR-RNAi*, adult-restricted *MIP-RNAi* also reduced sleep levels significantly in daytime and night-time (Figure S9D and S9E). With these results, we conclude that adult-specific expression of SPR and MIP is important for maintaining normal baseline sleep.

### MIP Downregulates cAMP in *pdf* Neurons Via SPR

Having shown that both SPR, and its ligand MIP, are essential for sleep maintenance and that wake-promoting *pdf* neurons are key *SPR* neurons, we next investigated whether MIP modulates the activity of *pdf* neurons through SPR. In the isolated brain, *pdf* neurons respond to bath-applied peptides, such as PDF and diuretic hormone 31, by upregulating intracellular cAMP [35]. As a GPCR, SPR can signal through two trimeric G protein pathways, Gα-i and Gα-o [18],[19], both of which downregulate intracellular cAMP upon activation. Therefore, we monitored MIP-induced cAMP dynamics in *pdf* neurons using Epac1-camps, a fluorescence resonance energy transfer (FRET)-based cAMP sensor [35],[36]. Upon binding to cAMP Epac1-camps undergoes a conformational change to increase inverse YFP/CFP FRET signal.

**Figure 4:**
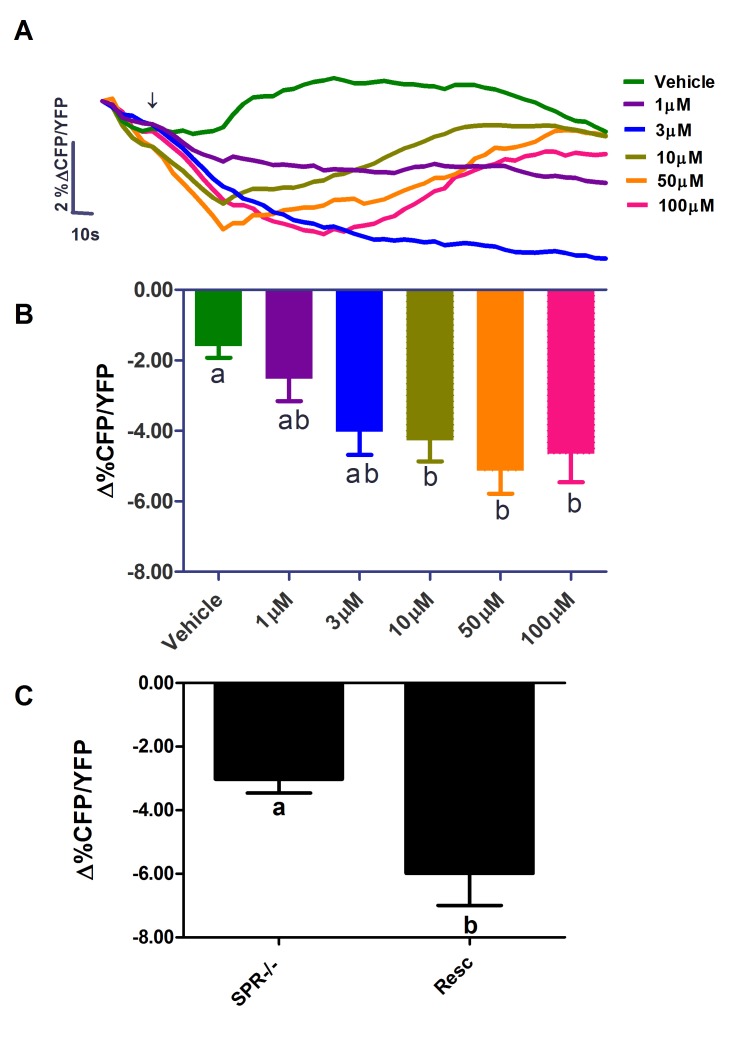
MIP modulates cAMP dynamics of l-LNvs through SPR. (A) Averaged Epac1-camps YFP/CFP FRET plots of l-LNvs from *pdf-Gal4,UAS-Epac1-camps* flies in response to various MIP doses applied as indicated by the arrow. The average drift in CFP/YFP ratio displayed by 28 untreated neurons from 11 brains, imaged identically to those treated with MIP and vehicle, were subtracted from the plots in (A) to correct for the effects of photo-bleaching. (B) A summary of the average maximum loss of Epac-1-camps CFP/YFP between 30 and 120 s for the data shown in (A). A one-way ANOVA revealed a significant effect of MIP concentration (*p*<0.0001) on the maximum loss of CFP/YFP ratio. A Dunn's multiple comparison test revealed significant differences (*p*<0.05) between vehicle controls and the 10, 50, and 100 µM MIP treatments. The sample sizes for (A) and (B) were as follows: for vehicle, 24 neurons from 14 brains (24, 14), 1 µM MIP (14, 8), 3 µM MIP (16, 3) 10 µM MIP (20, 11), 50 µM MIP (20, 10), and 100 µM MIP (12, 9). (C) The l-LNvs of *SPR*-deficient mutants (*SPR^−/−^,pdf-Gal4,UAS-Epac1-camp*) do not display an obvious loss of Epac1-camps CFP/YFP signal in response to 50 µM MIP (compare to B). However the rescue of SPR expression within the l-LNvs (*SPR^−/−^,pdf-Gal4,UAS-Epac1-camp,UAS-SPR*) results in wild-type like responses to MIP. An unpaired t-test revealed a significant difference between the effects of 50 µM MIP on SPR^−/−^ mutant and SPR rescued l-LNvs (*P* = 0.007). Sample sizes: SPR^−/−^ (15, 6), Rescue (11, 5). For (B) and (C) error bar represent SEM. The letters “a” and “b” in (B) and (C) denote significantly different groups.

Using *pdf-Gal4/UAS-Epac1-camps* females, we asked if the l-LNvs are responsive to synthetic MIP peptide. Bath perfusions of MIP at 10–100 µM resulted in significant reductions in inverse Epac1-camps FRET (henceforth, reduction of cAMP) in l-LNvs, compared to vehicle control (Figure 4A and 4B). Perfusions of MIP at 1 and 3 µM concentrations yielded inhibitory trends, but a comparison of maximal loss of inverse FRET did not reveal a statistically significant difference from vehicle controls. We also examined s-LNvs in the explanted brain. Like l-LNvs, s-LNvs also displayed inhibitory trends in response to MIP at concentrations higher than 10 µM; however, these were of lower magnitude and more variable compared to the responses of the l-LNvs (Figure S10).

Next, we asked whether MIP action on the l-LNvs occurs through SPR by examining MIP-induced cAMP dynamics in the l-LNvs of *SPR*-deficient mutants (*SPR^−/−^*; *pdf-Gal4/UAS-Epac1-camps*). Unlike wild-type neurons, mutant neurons did not display obvious reduction in cAMP in response to 50 µM MIP (Figure 4C). When SPR expression was rescued in SPR^−/−^ mutant l-LNvs, these neurons now responded to 50 µM MIP with a reduction in the cAMP (Figure 4C). These results support the hypothesis that MIP acts directly to reduce cAMP levels in *pdf* neurons through SPR.

In our *ex vivo* cAMP monitoring experiments, the minimum MIP concentration required to elicit statistically significant maximum Epac1-camps inverse FRET changes in l-LNvs neurons was ∼10 µM. This is substantially higher than the MIP concentration required for SPR expressed in cultured cells to start responding, which is around 1 nM. Because we did not remove neurolemma, a blood-brain barrier that separates bathing saline and the brain neurons, it was expected that much higher doses of peptides would be necessary to elicit a response detectable with live brain imaging. Similarly, *pdf* neurons expressing PDF receptor (PDFR) start to respond to micromolar concentrations of bath-applied PDF in the explanted brain [35], whereas PDFR in cultured cells can respond to PDF at a concentration as low as 0.1 nM [37],[38]. Furthermore, low doses of cAMP function mainly via the cAMP-dependent protein kinase A (PKA), whereas higher cAMP concentrations exert additional effects through Epac [39]. Indeed, in some case the Epac1-camps sensor failed to report low levels of cAMP, which a PKA-based cAMP sensor could reliably detect [40]. Thus, it is also possible that low concentrations of MIP modulate *pdf* neurons by reducing PKA activity without causing measurable FRET changes in the Epac1-camps cAMP sensor.

Since MIP appears to display some inhibitory effects on the s-LNvs, key pacemaker cells controlling the circadian rhythm [41],[42], we examined the circadian rhythm of flies lacking either SPR or MIP, but detected no obvious defect (Figure S11; Table S1). In addition, they also displayed intact morning anticipations both in LD and DD conditions, suggesting that the rather weak and variable SPR-MIP signaling within the s-LNvs is dispensable for pacemaker functions (Figure S11).

### Major MIP Secretion Occurs prior to and during Night-time Sleep

**Figure 5:**
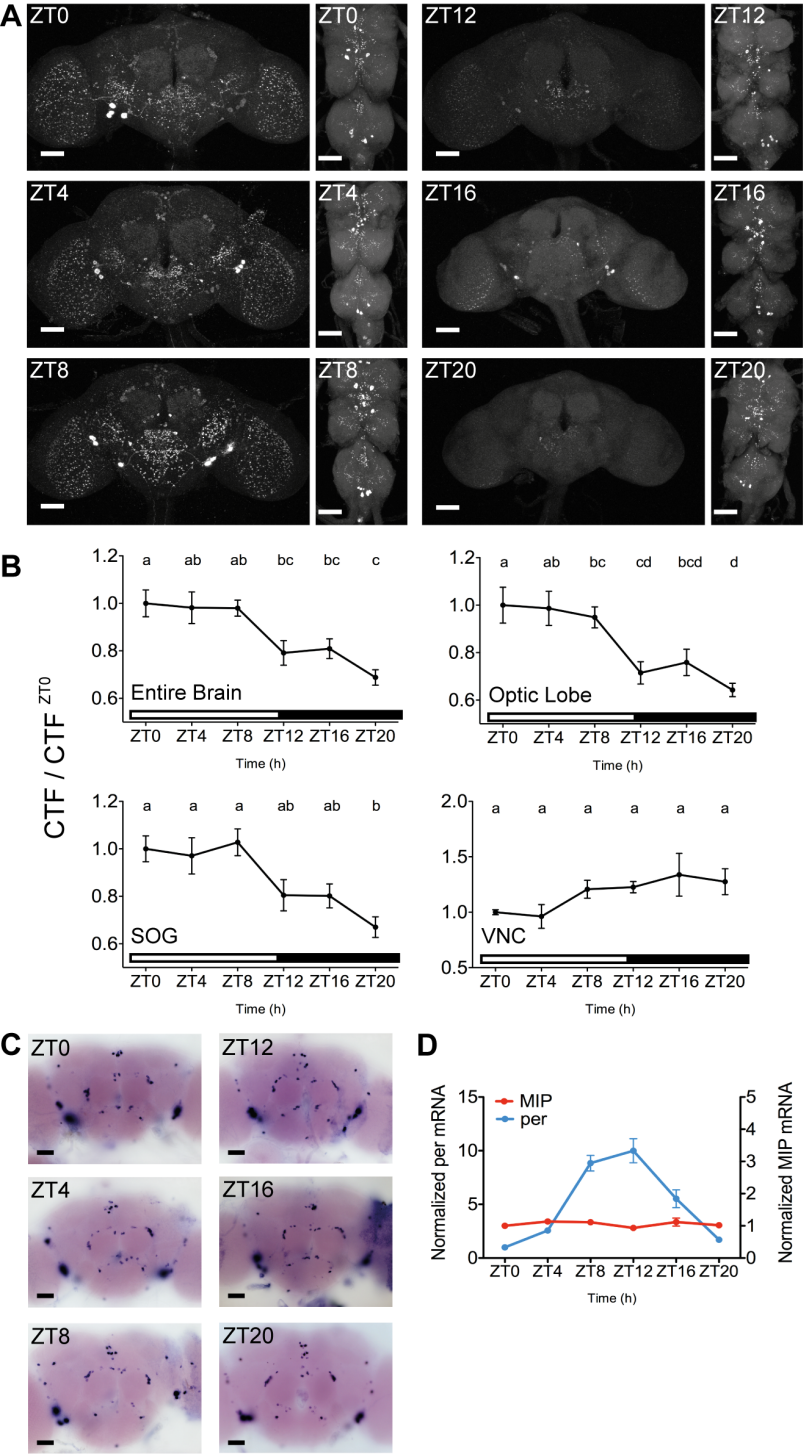
Comparisons between MIP protein and mRNA levels suggest synchronized and massive secretion of MIP occurring in the brain between ZT 8 and ZT 20. (A) Representative confocal images of anti-MIP staining of the *w^1118^* male brain (left) and the ventral nerve cord (VNC, right), isolated at six different time points (ZT 0, 4, 8, 12, 16, and 20). (B) Normalized MIP-immunoreactivity of the indicated CNS areas. *n* = 15–24 (for the brain regions); *n* = 5–6 (for the VNC). The letters from “a” to “d” indicate significant differences between ZT groups (*p*<0.05), determined by one-way ANOVA with Tukey's post hoc test. (C) Bright-field images of the male brain stained using *in situ* hybridization for MIP mRNA transcripts. Scale bars, 50 µm. (D) MIP (red) and per (blue) mRNA transcript levels in the male heads, measured by quantitative reverse transcription PCR and normalized to ZT 0. *n* = 3. Data are shown as means ± SEM.

Our results show that MIP is a sleep-promoting factor, which presumably reduces the excitability of wake-promoting *pdf* neurons by decreasing their intracellular cAMP levels. As a sleep regulator, MIP would be released prior to or during sleep. Thus, we used an anti-MIP antibody to measure MIP levels during normal sleep-wake cycles. Remarkably, the intensity of anti-MIP activity in the brain oscillates throughout the cycle. The oscillation is synchronized in most brain MIP neurons and processes (Figure 5A and 5B). The brain anti-MIP level increases early in the morning (zeitgeber time [ZT] 0) and stays elevated during daytime (ZT 0–8). Then, it drops markedly at ZT 12, prior to onset of the night-time sleep phase, and remains low during the night. The strongest reduction was detected at ZT 20 h, when the sleep stabilizing drive is most required (Figure 5B). Unlike the brain, the ventral nerve cord (VNC) showed no sign of anti-MIP oscillation (Figure 5B).

Next, we examined MIP mRNA levels in the brain using *in situ* hybridization. Unlike anti-MIP activities, the MIP transcript level does not oscillate and remains stable during the wake-sleep cycle (Figure 5C). This result was also confirmed by quantitative reverse transcription PCR experiments, which showed that MIP mRNA in the head remains constant throughout the day (Figure 5D). In contrast, the transcript levels of a central clock gene *period* changed during the cycle, and revealed the peak level at ZT 8 and 12. Because neuropeptides are packaged in large dense-core vesicles and transported to distal axon terminals slowly [43], it takes at least several hours to replenish the peptide vesicle pools after depletion [44]. Thus, the strong reduction of anti-MIP staining without measurable changes in transcript levels reflects massive secretory activity of the brain MIP neurons. To verify that the loss of anti-MIP staining is a consequence of prolonged neural activation, we used the *Drosophila* transient receptor potential A1 (dTrpA1), a warmth-activated cation channel, to confirm that thermal activation of MIP neurons depletes anti-MIP staining, almost completely (Figure S12). All together, these results strongly suggest that MIP neurons in the brain become active between ZT 8 and ZT 20, and secrete their peptidergic contents constitutively and synchronously, during which time the sleep drive is greater than any other time of the day.

### Sleep Deprivation Depletes MIP in Medulla

**Figure 6:**
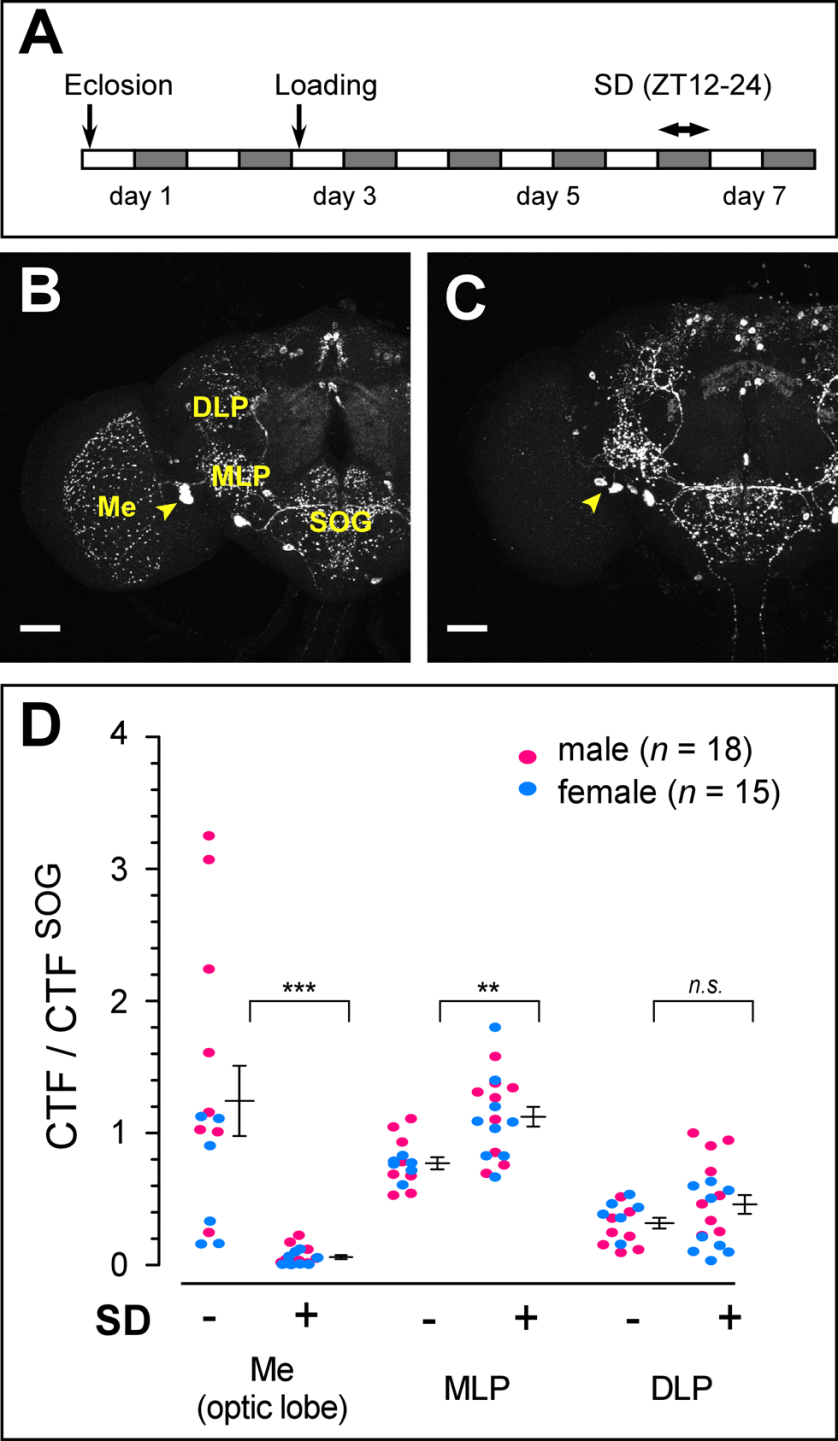
Sleep-deprived flies have reduced MIP levels in neurons innervating the optic lobe medulla. (A) Protocol for sleep deprivation (SD). (B, C) Representative confocal images of anti-MIP staining of the *w^1118^* female brain subjected to non-SD (B) and SD (C) conditions. Yellow arrowheads indicate somata of *MIP-LMIo*. (D) Normalized MIP-immunoreactivity of non-SD (−) versus SD (+) female and male flies in the indicated areas of interest. Data are shown as means ± SEM. **, *p*<0.01, ***, *p*<0.001 for the comparison by Student's *t* test. Scale bars, 50 µm. Me, medulla; DLP, dorso-lateral protocerebrum.

The marked reduction of the brain anti-MIP levels during sleep cycle is consistent with the release of MIP to promote sleep *in vivo*. To further explore this hypothesis, we asked whether sleep deprivation changes MIP levels in the brain. Since MIP works as a sleep-promoting factor and its level decreases during the sleep state, sleep deprivation should drive flies to release MIP continuously, and in consequence result in considerable loss of anti-MIP staining. Indeed, 12 h-long mechanical sleep deprivation significantly reduced anti-MIP labeling (Figure 6A–6C). The loss of anti-MIP labeling was particularly evident in axons of the lateral MIP-immunoreactive optic lobe (*LMIo*) neurons innervating the optic lobe medulla (Figure 6C), but less pronounced in axons of other *MIP* neurons arborizing the median lateral protocerebrum (MLP), dorso-lateral protocerebrum (DLP), and suboesophageal ganglion (SOG) (Figure 6C and 6D). A strong reduction of anti-MIP labeling in *LMIo* indicates massive secretion of MIP from this site during sleep deprivation.

We noted that anti-MIP staining in MLP was elevated moderately after sleep deprivation. The processes arborizing MLP come from *inferior contralateral interneurons* (*ICLI*), a pair of neurons that express natalisin (NTL), a tachykinin-like neuropeptide implicated in sexual activities of both sexes, as well as MIP (Figure S13A and S13B) [45]. Thus, we examined sleep functions of *ICLI* by suppressing MIP expression using *NTL-Gal4* driver, and found that *ICLI*-specific knockdown of MIP did not affect the baseline sleep architecture (Figure S13C and S13D). Nevertheless, the observation that sleep deprivation suppresses secretory activities of *ICLI* that are important for sexual behavior raises an intriguing possibility that *ICLI* may serve as a link between sleep and sexual activity.

### MIP and SPR Are Required for Sleep Homeostasis

**Figure 7:**
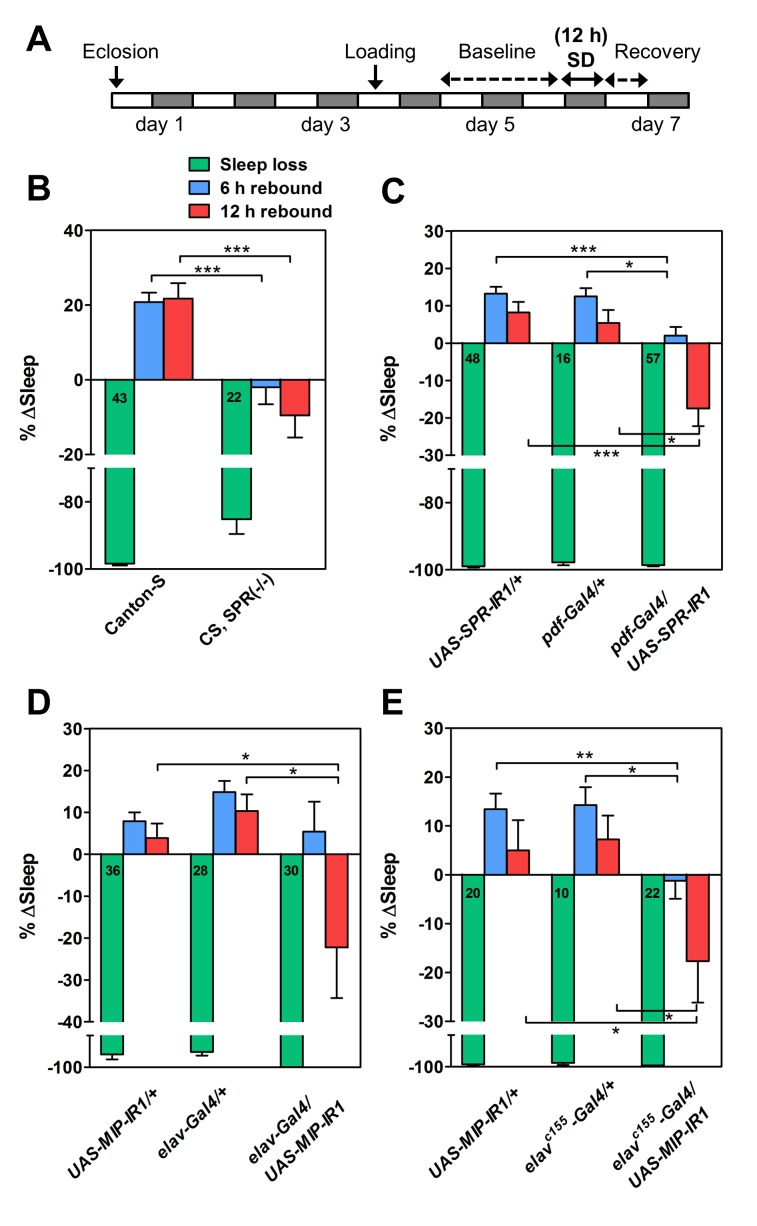
MIP and SPR are required for maintaining sleep homeostasis. (A) Experimental protocol used to quantify sleep homeostasis. (B–E) The percentage change in sleep (% Δsleep) after 12 h of mechanical sleep deprivation (green), 6 h (blue), and 12 h of sleep recovery (red) in females of indicated genotypes, measured the next morning. Number in bars indicates *n*. Data are shown as means ± SEM. *, *p*<0.05, **, *p*<0.01 ***, *p*<0.001 for the comparison to each controls by Student's *t* test.

Sleep is under the control of two regulatory systems, circadian and homeostatic, which define sleep timing and duration, respectively [46]. Since *MIP-LMIo* neurons deplete their contents in response to sleep deprivation, we suspected that MIP-SPR pathway may play a role in maintaining sleep homeostasis. To test this, the SPR deficient mutant and its isogenic control were subjected to 12 h-long mechanical sleep deprivation during the night (ZT 12–24), and allowed to recover the sleep loss in the following morning (Figure 7A). With this protocol, control flies showed a significant amount of sleep rebound (∼20% of lost sleep) after sleep deprivation. In contrast, the SPR deficient mutant showed no sleep rebound (Figure 7B). In parallel, we also observed lack of sleep rebound in *pdf* neuron-specific *SPR-RNAi* (*pdf-Gal4/UAS-SPR-IR1*), indicating that SPR expression particularly in *pdf* neurons is required for the normal sleep homeostasis (Figure 7C).

Next, we investigated sleep rebound of pan-neuronal *MIP-RNAi* flies. Compared to controls, *elav-Gal4/UAS-MIP-IR1* showed a significant reduction in sleep rebound when measured for 12 h, but not for 6 h (Figure 7D). The lack of phenotype in the 6 h rebound could be due to insufficient knockdown of MIP expression in this particular genotype. Thus, we adopted a stronger *elav^C155^-Gal4* driver, and confirmed that *elav^C155^-Gal4/UAS-MIP-IR1* flies had greatly attenuated sleep rebound for both the 6 and 12 h periods after sleep deprivation (Figure 7E). With these data, we conclude that MIP-SPR signaling pathway is important for the *Drosophila* sleep homeostasis.

### *MIP* Neurons and *pdf*-Positive *SPR* Neurons Arborize the Same Brain Region

**Figure 8:**
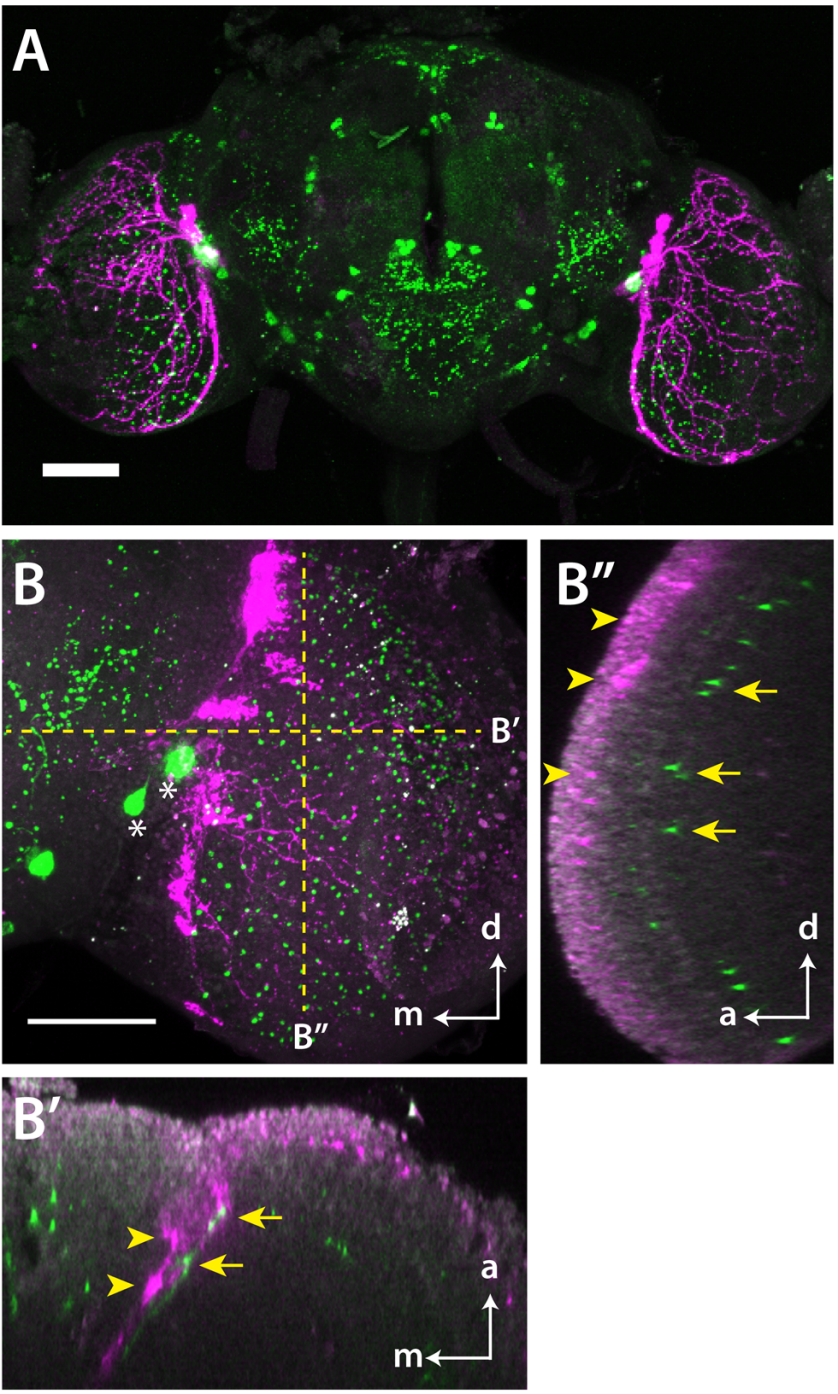
Axonal processes of *MIP-LMIo* neurons innervate the dendritic field of *pdf* neurons. (A) A confocal image of *pdf-Gal4/UAS-EGFP* fly brain stained with anti-MIP (green) and anti-EGFP (magenta) antibodies. The brain is oriented with dorsal up. (B) A high magnification confocal image of the optic lobe of *pdf-Gal4/UAS-DenMark* fly brain stained with anti-MIP (green) and anti-RFP (magenta) antibodies. Anti-RFP labels a dendrite marker, DenMark, which visualizes the dendritic field of *pdf* neurons. Asterisks indicate somata of *MIP-LMIo* neurons. Sections perpendicular to the dotted yellow lines are shown separately in (B′) and (B″). (B′) shows MIP-immunoreactive processes (yellow arrows) innervate the dendritic field of *pdf* neurons (yellow arrowheads). (B″) shows some MIP-labeling (arrows) occurs near the dendritic field of *pdf* neurons (arrowheads), indicating that MIP can also function as a paracrine factor. White arrows labeled with d, a, m indicate dorsal, anterior, and medial orientations, respectively. Scale bars, 50 µm.

Marked reduction of anti-MIP labeling in the medulla of sleep-deprived flies suggests that *LMIo* neurons are linked to sleep regulation. Furthermore, SPR-positive *pdf* neurons also arborize on the entire distal surface of the medulla (Figure 8A). To determine whether the dendritic field of *pdf* neurons contacts axonal processes of *LMIo* neurons, we prepared flies expressing a dendrite marker [47] in *pdf* neurons (*pdf-Gal4/UAS-DenMark*) and simultaneously visualized both the dendrite marker and MIP (Figure 8B). The dendrites of the *pdf* neurons are distributed around the base of the medulla and in a few locations closely apposed to MIP-positive processes of *LMIo* neurons (arrowheads and arrows in Figure 8B′). Overall, however, the staining pattern of *pdf* neuron dendrites and MIP-positive axonal varicosities in the medulla suggest that they are unlikely to make synaptic contacts (Figure 8B″). We propose, therefore, that MIP may act like many other neuromodulators via volume transmission [48]. In other words, it diffuses to activate SPR on the dendrites and somas of l-LNvs and s-LNvs.

## Discussion

Here we report the discovery of a peptidergic modulatory pathway particularly important in stabilizing sleep and maintaining sleep homeostasis in *Drosophila*. The key molecules in this novel sleep-regulating pathway are SPR and its peptide ligand MIP. SPR was first identified as a receptor that triggers PMR by mediating actions of the seminal protein SP in females [18]. Although previous biochemical studies demonstrated that SPR could interact with MIP as well as SP, there was no evidence that the interaction between MIP and SPR is biologically relevant in *Drosophila* [21]. By combining genetic analyses and optical activity imaging, we provide several independent lines of evidence demonstrating that MIP consolidates sleep state and maintain sleep homeostasis by acting through SPR expressed in arousal-promoting *pdf* neurons.

First, flies lacking either SPR or MIP have a highly similar sleep phenotype. Second, sleep phenotypes of MIP or SPR mutant are manifested regardless of sex, consistent with previous accounts that unlike SP, MIP and SPR expression in the brain show little sexual difference [18],[33]. Third, *ex vivo* optical activity imaging revealed that exogenous application of MIP downregulates cAMP levels in SPR-expressing *pdf* neurons, but not in SPR-deficient mutant neurons. Fourth, the sleep phenotypes of SPR-deficient mutants are rescued by restoring SPR expression with insect SPRs that are highly sensitive to MIP, but less sensitive to SP. Hence, SPR interacts with two evolutionarily unrelated sets of ligands, each of which controls completely different behaviors: SP for reproductive behaviors and MIP for sleep behavior. For sleep behaviors, all phenotypes observed in the SPR deficient mutant were also observed in *MIP-RNAi*. Thus, there is no reason to assume additional ligand(s) for SPR besides SP and MIP at this moment. Nevertheless, our finding that a peptide GPCR can mediate actions of multiple, unrelated groups of ligands should be taken into consideration in searching for peptides and/or other types of ligands for GPCRs.

**Figure 9:**
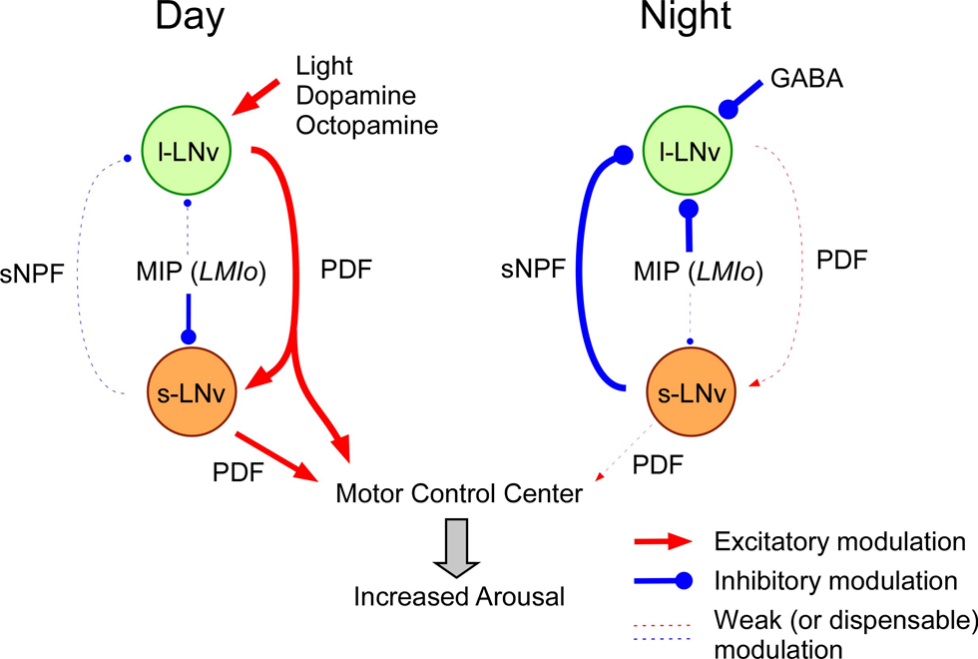
A model depicting an operating principle of three major metabotropic pathways that regulate sleep. During the day, light and modulatory inputs from other brain areas activate l-LNvs to secrete PDF, a major arousal-promoting factor. PDF from l-LNvs in turn activates the motor control center directly or indirectly through s-LNvs, and keeps flies awake. During the day, the sleep pressure gradually builds up and stimulates *MIP-LMIo* neurons to secrete MIP, which attenuates PDF release by modulating s-LNvs and allows the daytime sleep to ensue. Released MIP is expected to act on l-LNvs as well, but its effects are probably cancelled by direct excitatory inputs from light, octopamine, and dopamine. At the beginning of the night, waning excitatory inputs and elevating GABAergic input to l-LNvs (mainly through ionotropic GABA_A_ receptor) drive flies fall asleep (not shown in the model). Thereafter, three metabotropic pathways (sNPF, MIP, and GABA) stabilize and maintain sleep state during the night by supplying inhibitory modulations to l-LNvs, and keeping them from releasing PDF. *MIP-LMIos* function as a sleep-pressure sensor and adjust their secretory activities accordingly. As the night approaches the end, sleep pressure declines and metabotropic GABA input is needed.

Our genetic analyses demonstrated that the expression of SPR in three *Gal4* neural populations (*cry-Gal4*, *C929-Gal4*, and *pdf-Gal4*) is required and sufficient for wild-type levels of sleep maintenance. Furthermore, anti-SPR staining confirmed the SPR expression in two major subsets of *pdf* neurons, l-LNvs and s-LNvs. In particular, l-LNvs are common to all three *Gal4* populations [28],[30],[49]. Thus, the most parsimonious explanation of our results is that SPR in l-LNvs mediates a sleep-related MIP function. This rationale is also supported by previous reports. Firstly, l-LNvs respond to light and other modulatory cues and promote arousal by releasing PDF [25],[49], a major wake-promoting factor functionally analogous to vertebrate orexin/hypocretin [5],[11]. Secondly, excitation of l-LNvs suppresses night-time sleep [11],[50]. Third, l-LNvs are major targets of inhibitory GABA-GABA_A_ signaling, which promotes sleep both in flies and mammals [11],[12]. Fourth, blocking sNPF-mediated inhibitory input to l-LNvs impairs sleep stability particularly in night-time [32]. Finally, MIP signaling through SPR can down-regulate cAMP levels in l-LNvs. Together, these and our genetic data provide cogent support for a role for MIP signaling in stabilizing the sleep state by modulating l-LNvs activities through the SPR (Figure 9).

Another group of *pdf* neurons, s-LNvs are critical in timing the onset of morning behavior and are the key pacemaker cells controlling the circadian locomotor rhythm [41],[42]. Although the precise role of s-LNvs in sleep regulation remains less clear, previous reports implicated that s-LNvs regulate sleep mainly by relaying information from l-LNvs. In response to light and modulatory substances, such as dopamine and octopamine, l-LNvs secrete PDF, which in turn elevates cAMP levels in s-LNvs by activating the PDFR [49]. Consistent with the role of s-LNvs in sleep regulation, knockdown of PDFR in *pdf* neurons (presumably affecting the s-LNvs and not the l-LNvs) elevates total sleep [11]. Recently, it was shown that s-LNvs produce sNPF, which modulates l-LNvs and stabilizes sleep, particularly in night-time [32]. Here, we also report that SPR expression in s-LNvs is important for maintaining daily sleep architecture. Knockdown of SPR in s-LNvs reduced daytime sleep and its average bout duration, whereas knockdown of SPR in l-LNvs reduced night-time sleep and its average bout duration. Together with previous results, our observations suggest that s-LNvs are involved in sleep regulation, and that MIP-SPR signaling stabilizes sleep by modulating the activity of s-LNvs directly and indirectly through l-LNvs (Figure 9).

Our genetic and cAMP imaging results indicate that MIP regulates sleep as a ligand for SPR. Thus, it is also important to know whether MIP is secreted at biologically relevant times. The monitoring of levels of MIP peptide and mRNA at various time points in a day suggested that almost all brain MIP neurons release their contents synchronously from dusk to dawn, when the majority of flies fall and stay asleep. The rhythmic secretory activity of MIP neurons is likely to be under the control of the circadian clock rather than environmental light, because initiation and termination of the MIP secretion occurs prior to the light-off (ZT 12) and the light-on time (ZT 24), respectively. Since MIP neurons are not part of the circadian clock network [33], it would be interesting to see in the future to elucidate how they interact with the neuronal circadian clock network.

Our results indicate that MIP release in most brain neurons appears synchronized, and MIP neurons in the brain arborize in many areas of the brain, including the olfactory glomeruli, the SOG, the lateral ventral protocerebrum, mushroom body, and so on (for further evidence, see [24],[33],[45]). Considering SPR is expressed broadly in large numbers of the brain neurons [18], massively secreted MIP in these sites probably modulates not only neurons important for locomotor activities and but also many others involved in diverse biological processes, such as olfactory, feeding, sexual activity, learning, and memory.

Like in the human situation, sleep in *Drosophila* is also affected by other behavioral aspects, such as stress, social interactions, learning, diet, feeding, and reproduction [14]. In females, mating suppresses daytime sleep, and male-derived SP is responsible for this sleep modulation [15]. On the other hand, SP also plays key roles in eliciting the PMR, such as reduced receptivity to further mating and increased egg-laying [18]. In this study, we clearly demonstrated that the sleep-relevant SPR circuits (l-LNvs and s-LNvs) are distinct from the PMR-relevant SPR circuit (*ppk^+^ fru^+^* neurons). Intriguingly, however, SP circulates in the haemolymph of mated females [51],[52], raising the possibility that the haemolymph-born SP activates SPR in the sleep circuit and modulates sleep. This is certainly a plausible scenario, considering that SP is a potent agonist for the SPR [18], and bath-applied SPR agonist (in this case, MIP) can affect cAMP levels in s-LNvs. In theory, however, the SPR activation in the sleep circuit either by haemolymph-born SP or centrally released MIP should promote sleep, rather than suppress it. Thus, we suspect that the daytime sleep loss observed in the mated female is not due to direct modulation of the SPR-sleep circuit by SP. Rather, SP actions on the PMR circuit elevate reproductive drives in mated females, which in consequence makes them spend more time during the day searching for food and egg-laying sites, and less time in falling asleep. Nevertheless, we cannot formally exclude the possibility that SP modulates female sleep. In theory, SP circulating in haemolymph of the mated female can promote sleep drive and counter wakefulness driven by reproductive motivations. Thus, we speculate that SPR may serve as a molecular integrator that computes reproductive-state coding signal (SP) and sleep-pressure coding signal (MIP) and therefore contribute to shaping daily sleep architecture.

Multiple lines of evidence indicate that MIP-SPR signaling is a part of the homeostatic control system. First, mutants lacking either MIP or SPR show significant reduction in total amount of sleep, which is an indicator of homeostatic regulation [5]. Second, sleep deprivation drives *MIP-LMIo*, a subset of brain MIP neurons to release MIP into the optic lobe medulla where *pdf* neurons innervate. We propose that *MIP-LMIo* senses sleep pressure and modulates MIP secretion to maintain optimum sleep duration. Lastly and most importantly, mutants lacking either MIP or SPR show no sleep rebound after sleep deprivation. Together, these observations suggest that the activity of MIP neurons is controlled by two separable pathways; one associated with the circadian clock network (see above), and the other associated with a sleep homeostat.

It has been proposed in mammals that activity-dependent metabolites, such as adenosine, GABA, prostaglandins, and cytokines, are involved in sleep homeostasis, particularly the sleep initiation phase [53]. The role of GABA signaling in sleep is conserved both in mammals and flies [5]. In *Drosophila*, GABA regulates both sleep initiation and maintenance because silencing GABAergic neurons results in a significant decrease of sleep latency from lights-off as well as mean sleep-bout duration [11],[13]. At the beginning of the night, GABA initiates sleep by inhibiting the activities of wake-promoting *pdf* neurons through the GABA_A_ receptor, a ligand-gated Cl^−^ channel [11],[12]. After animals fall asleep, at least three modulatory pathways stabilize the sleep state and sustain it throughout the night (Figure 9): sNPF-sNPF receptor [32], GABA-GABA_B_ receptor 2 [54], and MIP-SPR (this study). All three pathways feed inhibitory modulation into l-LNvs, and in consequence keep these neurons from releasing PDF during the night (Figure 9). Unlike the other two pathways, MIP-SPR signaling is also important for stabilizing daytime sleep. Our model predicts that in the morning, shortly before light-on, s-LNvs release less sNPF than PDF. This probably is due to faster depletion of sNPF pool in s-LNvs during the night, as suggested by the fact that sNPF mRNA levels in s-LNvs are 30-fold higher in the morning (ZT 0) than in the evening (ZT 12) [31]. Then, subsequent to light-on l-LNvs are stimulated to release PDF [55], which in turn modulates the motor control centers either directly or indirectly through s-LNvs [11],[50], and in consequence promotes wakefulness. Later, as sleep pressure builds up during the day, *MIP-LMIos* sense the sleep pressure and release MIP, allowing sleep to ensue in the middle of the day. MIP is expected to act via volume transmission, meaning that once released, it can access both l-LNvs as well as s-LNvs. In daytime, however the inhibitory actions of MIP on l-LNvs are fully countered by excitatory inputs from environmental light via dopamine and octopamine signaling, partly because MIP secretion is weaker at this time of day than at night-time. For siesta sleep, therefore SPR activation in s-LNvs is more important than that in l-LNvs (Figure 9).

Several lines of evidence indicate that MIP, not SP, is the ancestral ligand of SPR [21],[23]. MIP can activate SPRs from diverse species including the sea slug *Aplysia*, whereas SP can only activate SPRs from *Drosophila* species at physiological levels. MIPs are also more potent than SP as SPR agonists. Furthermore, orthologs of *SPR* and *MIP* are clearly detectable in most (but not all) sequenced genomes from Lophotrochozoa and Ecdysozoa. By contrast, *SP* has been found only in the genomes of a few closely related *Drosophila* species, indicative of their recent origin. Together, these observations suggest that the SPR-MIP signaling axis is evolutionarily ancestral, whereas the SPR-SP signaling axis arose only recently in *Drosophila* evolution, concomitantly with the emergence of SP. Our discovery that sleep regulation is a possible ancestral SPR function is a critical step forward in understanding how the SPR evolved functional multiplicity by recruiting a newly emerging ligand.

## Materials and Methods

### Animals

Flies were reared on standard food containing dextrose, cornmeal, and yeast at room temperature. *UAS-AeaSPR*, *UAS-BomSPR*, and *UAS-TrcSPR* were generated as described previously [18]. When expressed in CHO cells, the TrcSPR was insensitive to both SP and MIP, tested at 10 µM (not shown). Each receptor coding sequence was cloned into the pPT13 vector (a custom-designed vector modified from a standard UAS vector) and inserted into a specific second chromosome site (defined here as VIE-72a) using the ΦC31 system [56]. Mutants and transgenic lines described previously are as follow: *CS*, *Df(1)Exel6234* (*SPR* ^−/−^), *UAS-SPR-IR1* and *UAS-DrmSPR* [18], *UAS-MIP-IR1* and *UAS-MIP-IR2* [21], *elav-Gal4* (III) [57], *UAS-Dicer2* [58], *cry-Gal4* [28], *pdf-Gal4* [30], *C929-Gal4* [29], *fru^Gal4^* [26], *tdc-Gal4* [59], *th-Gal4* [60], *ort-Gal4* [61], *C232-Gal4*, *C161-Gal4* and *189Y-Gal4* [62], *201Y-Gal4* [63], *1471-Gal4* [64], *C309-Gal4* [63], *OK348-Gal4* [65], and *UAS-DenMark* [47]. The *elav^C155^-Gal4* (I) and *elav-Gal4* (II) was obtained from the Bloomington *Drosophila* Stock Center (Bloomington stock number, 8760 and 8765). The *elav-Gal4* (III) carrying a *UAS-Dicer2* on the X chromosome was used for all pan-neural *RNAi* experiments, unless stated otherwise.

### Sleep Assay

Virgin females and males were collected at eclosion, and aged for 5 days in groups of 10–15 before assay. Mated females were prepared by combining 2–3-day-old virgin females with males for at least 2 days. Five-day-old flies were loaded in 65 mm×5 mm glass tubes containing 4% sucrose and 2% agar, and their 1 min-bin locomotor activity was collected with DAM System monitors (Trikinetics) in an incubator at 25°C and 60% humidity. Flies were monitored for 6 days under a 12 h light∶dark (LD) cycle. To compute sleep parameters, the data from days 5 and 6 were analysed with custom-built software. An uninterrupted inactivity lasting at least 5 min was counted as a sleep bout [7]. Flies with no activity in the final day of analysis were removed.

### Optical cAMP Imaging

The measurement of relative cAMP levels within single neuron cell bodies during bath application of MIP was done as previously described [35] with minor modifications. Flies for imaging experiments were reared at 25°C under a 12∶12 light-dark cycle. Living brains expressing the cAMP sensor Epac1-camps in neurons of interest were dissected from 3- to 5-day-old flies into room temperature hemolymph-like saline (HL3) consisting of (in mM): 70 NaCl, 5 KCl, 1.5 CaCl_2_, 20 MgCl_2_, 10 NaHCO_3_, 5 trehalose, 115 sucrose, 5 HEPES; pH 7.1 [66] and stuck to the bottom of 35×10 mm Falcon Petri Dishes containing a petri dish perfusion insert containing 360 µl HL3 (Bioscience Tools). Dissections were always performed between 6 and 11 hours after lights-on. Brains were allowed to settle and adhere to the bottom of the dish for 10 minutes before imaging. Epac1-camps imaging of relative cAMP levels was conducted as previously described [67]. Briefly, frames containing LNv cell bodies were scanned as single optical sections once every five seconds. After 30 s of initial scanning, 40 µl of MIP peptide (at 10× the target concentration in 1% DMSO) was gently added into the perfusion insert by hand with a micropipette to yield the target MIP concentration and 0.1% DMSO. cAMP responses of neurons of interest were monitored for a total of 5 min with an Olympus Fluoview 1000 confocal microscope equipped with the Fluoview software (Olympus). Post-imaging analysis of Epac1-camps responses was done as previously described [68]. HPLC-purified synthetic MIP4 (EPTWNNLKGMW-amide) was obtained from AnyGen.

### Sleep Deprivation and Sleep Rebound

For sleep deprivation, 3-day-old flies were individually loaded into 65-mm×5-mm glass tubes containing food, and entrained to a 12 h LD cycle. After 3 days of entrainment, flies were subjected to the sleep deprivation protocol described [69] previously during the dark cycle (ZT 12–24) at day 4. For the behavioral sleep homeostasis assay, flies were subjected to the SNAP-based sleep deprivation described previously [70] and allowed to recover the sleep loss in the following morning (day 7) [71].

### Immunohistochemistry and In Situ Hybridization

For SPR staining, the brains were dissected under ice-cold PBS (pH 7.4) and fixed in PBS containing 4% paraformaldehyde at 4°C. Note that it was crucial to fix the tissue for more than 24 h for the successful anti-SPR staining [18]. After washing and blocking, the brain tissues were incubated in an anti-SPR antibody (1∶500) for 48 h at 4°C and then with an HRP-conjugated goat anti-rabbit antibody (1∶100; Invitrogen catalogue number T20924) for 1 h at RT. Then, the brain was stained with Tyramide signal amplification kit (Invitrogen) according to the manufacturer's instruction, and mounted in Vectashield (Vector Labs).

For MIP and DenMark labelling, brains were processed similarly, but fixed for 30 min at RT. Mouse monoclonal anti-MIP antibody 1A4 (1∶1,000) [72] and an Alexa-488 goat anti-mouse (1∶1,000; Invitrogen catalogue number A11001) were used as primary and secondary antibodies, respectively. To visualize DenMark, an anti-RFP antibody (1∶2,000; Invitrogen catalogue number R10367) and an Alexa-568 goat anti-rabbit antibody (1∶2,000; Invitrogen catalogue number A11011) were used. Images were acquired with a Zeiss LSM700/Axioscope laser scanning microscope and processed using ImageJ [73]. For quantification of anti-MIP labeling, labeling intensity was measured as the corrected total fluorescence (CTF), calculated by subtracting the integrated fluorescence of an area of interest with its background fluorescence. Because MIP-immunoreactivity in the subesophageal ganglion (SOG) area were not different between treatments, CTF of each brain area were normalized by dividing CTF of the SOG.

In situ hybridization was performed as described [72]. Dissected brains were fixed in 4% paraformaldehyde for overnight at 4°C, stored in 70% ethanol, washed with PBS with Tween-20, treated with proteinase K and glycine, and hybridized with a digoxygenin (DIG)-labeled single-stranded DNA probe for overnight at 48°C. Blocked with 1% BSA, the brains were incubated with anti-DIG antibody conjugated with alkaline phosphatase (Roche) for overnight at 4°C, and stained with NBT-BCIP (Roche). Primers used to generate MIP probe was a forward primer (5′-CTGATGGTGCTCCTCATCCT-3′) and a reverse primer (5′-CTGTGCTACGGCGATTCTCT-3′). Images were taken in a bright field microscope (Olympus BX53).

## Supporting Information

Figure S1 **Mating status of females makes little difference in sleep architecture (related to** **Figure 1** **).** (A, G) Standard sleep plots of wild-type *CS* (A) and *w^1118^* (G) females in a 12-h∶12-h light∶dark cycle (L∶D). Sleep parameters of virgin (v) and mated (m) females are compared in *CS* (B–F) and *w^1118^* (H–L) control strains. Tested females were age-matched. Note that mated females were examined at least 4–6 days after mating (see experimental procedures). (B, H) Daytime sleep duration. (C, I) Night-time sleep duration. (D, J) Waking activity. (E, K) Sleep bout number per day. (F, L) Mean sleep-bout duration. Number in bars indicates *n* of the tested flies. Data are shown as means ± SEM. All comparisons between virgin and mated females are not significant (*p*>0.05, Student's *t* test and Mann-Whitney U test).
(TIF)

Figure S2 **Mutants lacking either SPR or MIP show reduced sleep in both light-dark (LD) and constant dark (DD) conditions (related to** **Figures 1** **and** **3** **).** (A–D) Standard sleep plots of indicated genotypes of females (A, C) and males (B, D) in a 12-h∶12-h light∶dark (LD) condition for 2 days and constant darkness (DD) for the subsequent 3 days. Shaded boxes depict dark periods. Diurnal and nocturnal sleep durations of indicated genotypes in LD (A′–D′) and DD condition (A″–D″). Number in parentheses or bars indicates *n* of the tested flies. Data are shown as means ± SEM. *, p<0.05; **, *p*<0.01; ***, *p*<0.001 for the comparison to its controls by Student's *t* test.
(TIF)

Figure S3 **Like SPR deficient mutants, pan-neural** ***SPR-RNAi*** **flies also show defects in sleep maintenance (related to** **Figure 2** **).** (A) Standard sleep plots of pan-neural *SPR-RNAi* (*elav-Gal4, UAS-SPR-IR1*) and its control females in a 12-h∶12-h light∶dark cycle (L∶D). Shaded boxes depict dark periods. (B–E) Sleep parameter of females of indicated genotypes. (F–I) Sleep parameter of males of indicated genotypes. (B, F) Total sleep duration per day. (C, G) Waking activity. (D, H) Sleep bout number per day. (E, I) Mean sleep-bout duration. Number in bars indicates *n* of the tested flies. Data are shown as means ± SEM. **, *p*<0.01; ***, *p*<0.001 for the comparison to both *Gal4* and *UAS* controls by Student's *t* test (B–D, F–H) and Mann-Whitney U test (E, I).
(TIF)

Figure S4 **SPR overexpression alone in wild-type background does not elevate baseline sleep (related to** **Figure 2** **).** (A–D) Standard sleep plots of virgin female (A, C) and males (B, D) of indicated genotypes. Shaded boxes depict dark periods. (A′–D′) Diurnal and nocturnal sleep durations of virgin females (A′, C′) and males (B′, D′) of indicated genotypes. Number in parentheses or bars indicates *n* of the tested flies. Data are shown as means ± SEM. All the comparisons to *Gal4* and *UAS* controls are not significant (*p*>0.05, Student's *t* test).
(TIF)

Figure S5 **SPR expression in l-LNvs and s-LNvs is important for nocturnal and diurnal sleep, respectively (related to** **Figure 2** **).** (A, F) Standard sleep plots of indicated genotypes of virgin females in a 12-h∶12-h light∶dark cycle (L∶D). Black bars in x-axis depict dark periods. (B, G) Daytime (ZT 0–12) sleep duration of indicated genotypes. (C, H) Night-time (ZT 12–24) sleep duration of indicated genotypes. (D, I) Average daytime (ZT 0–12) sleep-bout duration of indicated genotypes. (E, J) Average night-time (ZT 12–24) sleep-bout duration of indicated genotypes. Number in parentheses or bars indicates *n* of the tested flies. Data are shown as means ± SEM. **, *p*<0.01; ***, *p*<0.001 for the comparison to both *Gal4* and *UAS* controls by Student's *t* test (B–C, G–H) and Mann-Whitney U test (D–E, I–J). Dataset used for Figure 2A is reanalysed.
(TIF)

Figure S6 **Like** ***UAS-MIP-IR1*** **,** ***UAS-MIP-IR2*** **combined with** ***elav-Gal4*** **also shortens total sleep by impairing sleep maintenance (related to** **Figure 3** **).** (A) Standard sleep plots of pan-neural *MIP-RNAi* (*elav-Gal4, UAS-MIP-IR2*) and its control females in a 12-h∶12-h light∶dark cycle (L∶D). Shaded boxes depict dark periods. (B–E) Sleep parameter of females of indicated genotypes. (F–I) Sleep parameter of males of indicated genotypes. (B, F) Total sleep duration per day. (C, G) Waking activity. (D, H) Sleep bout number per day. (E, I) Mean sleep-bout duration. Number in parentheses or bars indicates *n* of the tested flies. Data are shown as means ± SEM. **, *p*<0.01; ***, *p*<0.001 for the comparison to both *Gal4* and *UAS* controls by Student's *t* test (B–D, F–H) and Mann-Whitney U test (E, I).
(TIF)

Figure S7 **Anti-MIP staining is greatly attenuated in two** ***MIP-RNAi*** **lines (related to** **Figures 3** **and S6).** The brain anti-MIP staining of *elav-Gal4 UAS-MIP-IR1* (A), *UAS-MIP-IR1* (B), *elav-Gal4 UAS-MIP-IR2* (C), and *UAS-MIP-IR2* (D). Scale bars, 50 µm.
(TIF)

Figure S8 ***Drosophila*** **SPR can be replaced with insect SPRs less sensitive to SP (related to** **Figure 4** **).** (A, B) Total sleep duration per day of virgin females (A) and males (B) of indicated genotypes. *AeaSPR*, *BomSPR*, and *TrcSPR* indicate *SPRs* from a mosquito *A. aegypti*, a moth *B. mori*, and a beetle *Tribolium castaneum*, respectively. Note that AeaSPR and BomSPR have strong sensitivity toward MIP, but intermediate or low sensitivity toward SP (for details, see text). TrcSPR, insensitive to either MIP or SP is used as a control. Number in parentheses indicates *n* of the tested flies. Data are shown as means ± SEM. **, *p*<0.01; ***, *p*<0.001 for the comparison to *w^1118^* control by Student's *t* test.
(TIF)

Figure S9 **Adult-specific knockdown of SPR or MIP reduces diurnal and nocturnal sleep in both sexes (related to** **Figures 1** **and** **3** **).** (A) Protocol for behavioral experiments in (B–E). RU486 treatment activates Gal4 expression in flies carrying *GeneSwitch-Gal4*. (B–E) Standard sleep plots of virgin females (B, D) and males (C, E) of indicated genotypes. (B′–E′) Diurnal and nocturnal sleep durations of virgin females (B′, D′) and males (C′, E′) of indicated genotypes. ‘+’ and ‘−’ indicate RU486 and vehicle treatment, respectively. Numbers in parentheses or bars indicate *n* of the tested flies. Data are shown as means ± SEM. *, *p*<0.05; **, *p*<0.01; ***, *p*<0.001 for the comparison between RU486 and vehicle by Student's *t* test.
(TIF)

Figure S10 **The effects of MIP on cAMP dynamics within the s-LNvs.** (A) Averaged Epac1-camps YFP/CFP FRET plots of s-LNvs from *pdf-Gal4,UAS-Epac1-camps* flies in response to 10 and 50 µM MIP doses applied as indicated by the arrow. (B) A summary of the average maximum loss of Epac-1-camps CFP/YFP for the data shown in (A) between 30 and 120 s. A one-way ANOVA revealed no significant effect of MIP concentration for the s-LNvs (*p*<0.1290) on maximum loss of CFP/YFP ratio. A Dunn's multiple comparison test revealed no significant differences (*p*>0.05) between vehicle controls and the 10, 50 µM MIP treatments. The sample sizes for (A) and (B) were as follows: for vehicle, ten neurons from nine brains (10, 9), 10 µM MIP (13, 8), 50 µM MIP (14, 11).
(TIF)

Figure S11 **Circadian activities of flies lacking either SPR or MIP are normal.** Average activity profiles in LD (left), and DD (middle) conditions, and average actograms throughout the behavioral analysis (right) of indicated genotypes. Note none of tested lines show obvious defects in circadian rhythms, morning and evening anticipations both LD and DD condition. Alternating white and black bar in the x-axis indicates LD cycle (12-h∶12-h), whereas gray and black bars indicate DD cycle.
(TIF)

Figure S12 **Activation of MIP neurons results in complete depletion of anti-MIP labeling in the brain (Related to** **Figure 5** **).** (A) The experimental protocol. Five-day-old males were subjected to thermo-activation at 30°C from ZT 12 to ZT 24, and their CNS were dissected and processed shortly thereafter. (B, C) The anti-MIP stained brain of *MIP-Gal4 UAS-dTrpA1* (B) or *UAS-dTrpA1* control males (C) subjected to the thermal activation. Scale bars, 50 µm.
(TIF)

Figure S13 **MIP expression in** ***MIP-ICLI*** **is not important for sleep regulation (related to** **Figure 6** **).** (A, B) The brain of *NTL-Gal4 UAS-MIP-IR1* (A) or *NTL-Gal4* control males (B) stained with anti-MIP. Note MIP expression in the MLP and SOG is greatly attenuated in the MIP-RNAi targeted by *NTL-Gal4*, confirming *MIP-ICLI* neurons (arrows) innervating the MLP and SOG express *NTL-Gal4*. (C, D) Total daily sleep duration of females (C) and males (D) of indicated genotypes. Number in bars indicates *n* of the tested flies. Data are shown as means ± SEM. Not significant (*p*>0.05) for the comparison to both *Gal4* and *UAS* controls by one-way ANOVA with Tukey's post hoc test. Scale bars, 50 µm.
(TIF)

Table S1 **Circadian rhythm parameters of SPR and MIP mutants and RNAi.**
(DOCX)

Text S1 **Supplemental materials and methods.**
(DOCX)

## Abbreviations

AeaSPR: *Aedes aegypti* SPR
BomSPR: *Bombyx mori* SPR
CNS: central nervous system
CS: Canton-S
DD: dark-dark
DrmSPR: Drosophila SPR
dTrpA1: Drosophila transient receptor potential A1
FRET: fluorescence resonance energy transfer
GPCR: G-protein coupled receptor
ICLI: inferior contralateral interneuron
l-LNv: large lateral ventral neuron
LD: light-dark
LMIo: lateral MIP-immunoreactive optic lobe
MIP: myoinhibitory peptide
MLP: median lateral protocerebrum
NTL: natalisin
pdf: pigment dispersing factor
PDFR: pigment dispersing factor receptor
PMR: post-mating behavioral responses
s-LNv: small lateral ventral neuron
sNPF: short neuropeptide F
SOG: suboesophageal ganglion
SP: sex peptide
SPR: sex peptide receptor
TrcSPR: *Tribolium castaneum* SPR
UAS: upstream activation sequence
ZT: zeitgeber time
