# Supplementary figures and images for: A Homeostatic Sleep-Stabilizing Pathway in Drosophila Composed of the Sex Peptide Receptor and Its Ligand, the Myoinhibitory Peptide
Source: PLoS Biol. 2014 Oct 21;12(10):e1001974. doi: 10.1371/journal.pbio.1001974 (PMC4204809; doi:10.1371/journal.pbio.1001974)

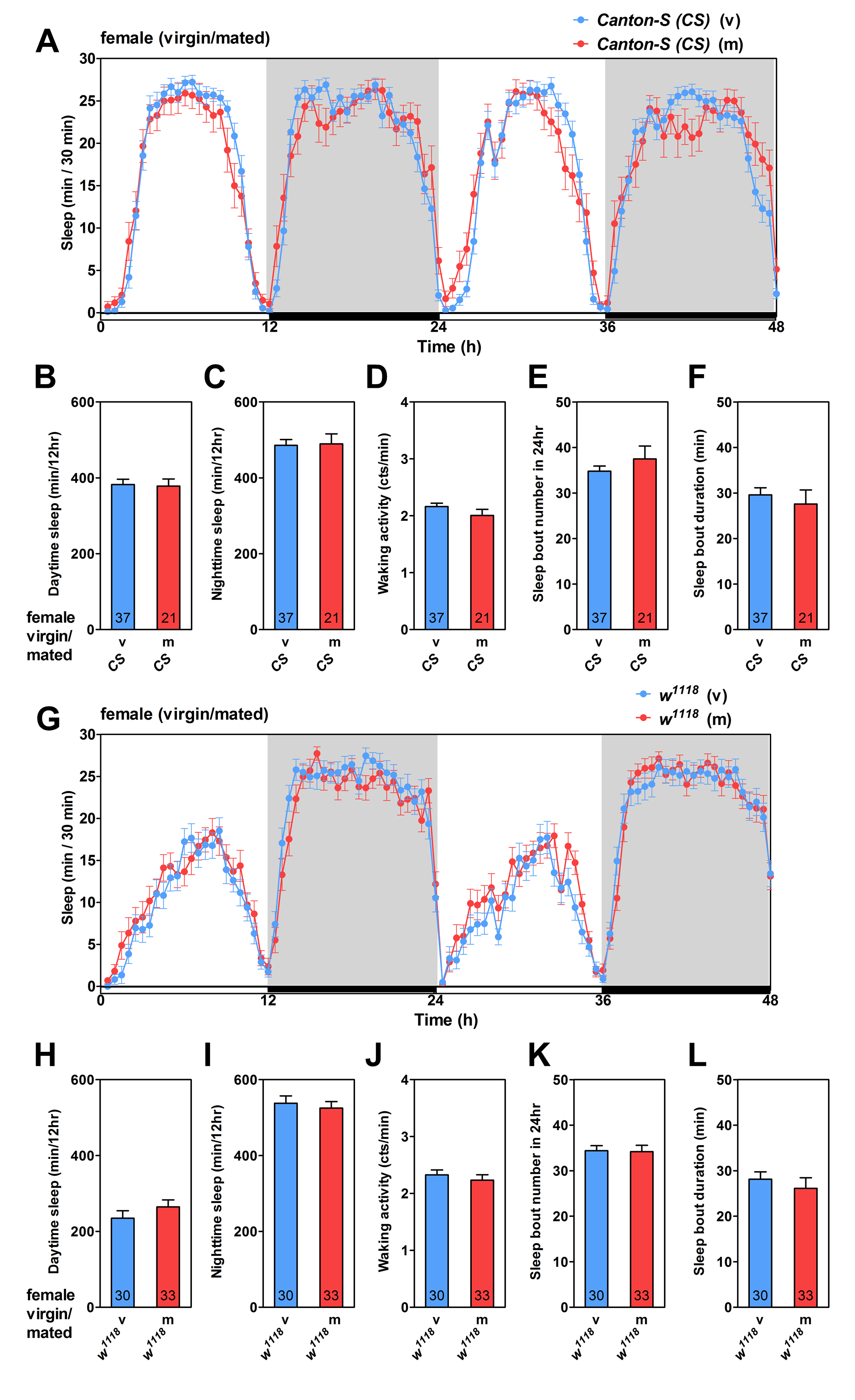

Supplement: Figure S1 — Mating status of females makes little difference in sleep architecture (related to Figure 1 ). (A, G) Standard sleep plots of wild-type CS (A) and w1118 (G) females in a 12-h∶12-h light∶dark cycle (L∶D). Sleep parameters of virgin (v) and mated (m) females are compared in CS (B–F) and w1118 (H–L) control strains. Tested females were age-matched. Note that mated females were examined at least 4–6 days after mating (see experimental procedures). (B, H) Daytime sleep duration. (C, I) Night-time sleep duration. (D, J) Waking activity. (E, K) Sleep bout number per day. (F, L) Mean sleep-bout duration. Number in bars indicates n of the tested flies. Data are shown as means ± SEM. All comparisons between virgin and mated females are not significant (p>0.05, Student's t test and Mann-Whitney U test). (TIF) [file pbio.1001974.s001.tif]

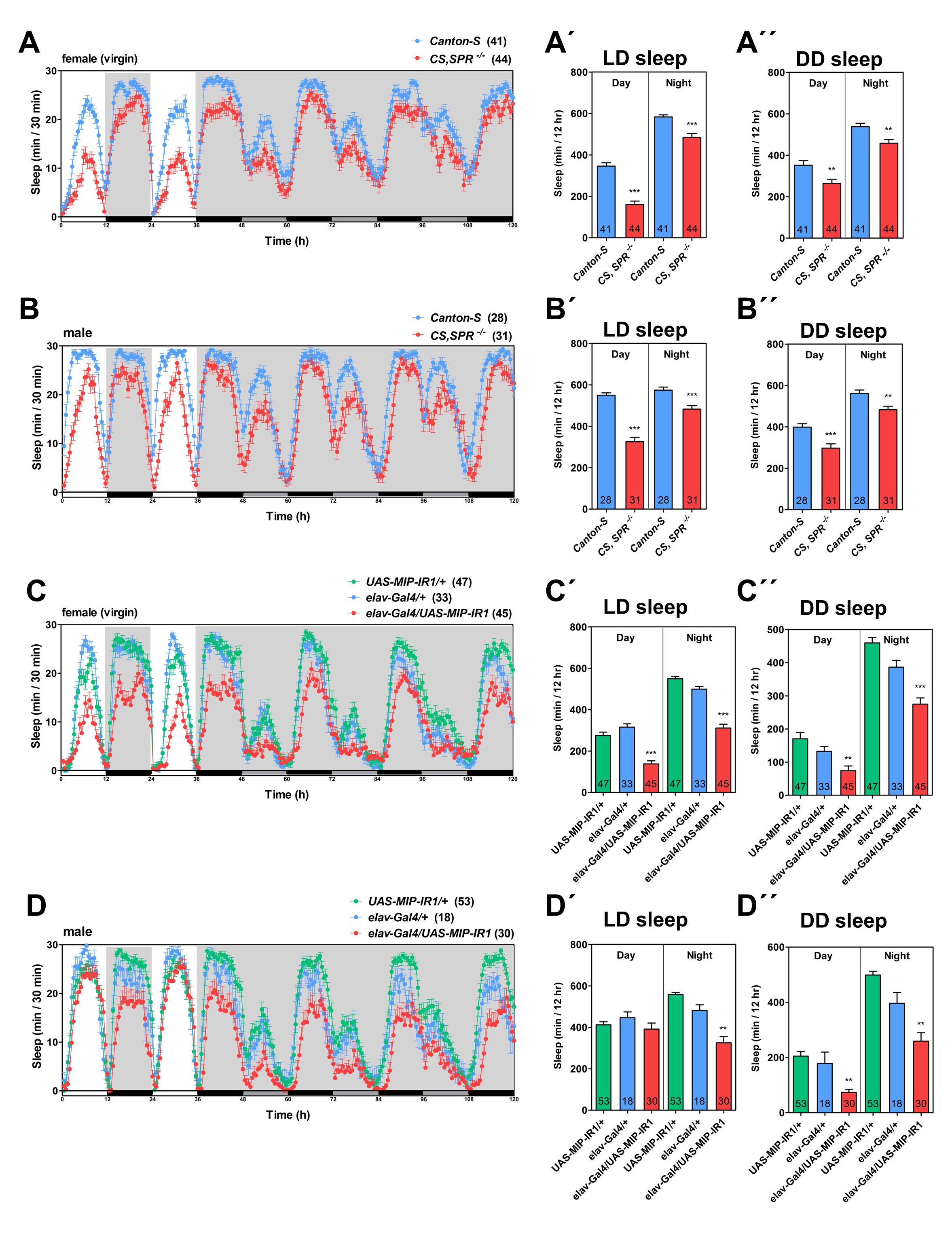

Supplement: Figure S2 — Mutants lacking either SPR or MIP show reduced sleep in both light-dark (LD) and constant dark (DD) conditions (related to Figures 1 and 3 ). (A–D) Standard sleep plots of indicated genotypes of females (A, C) and males (B, D) in a 12-h∶12-h light∶dark (LD) condition for 2 days and constant darkness (DD) for the subsequent 3 days. Shaded boxes depict dark periods. Diurnal and nocturnal sleep durations of indicated genotypes in LD (A′–D′) and DD condition (A″–D″). Number in parentheses or bars indicates n of the tested flies. Data are shown as means ± SEM. *, p<0.05; **, p<0.01; ***, p<0.001 for the comparison to its controls by Student's t test. (TIF) [file pbio.1001974.s002.tif]

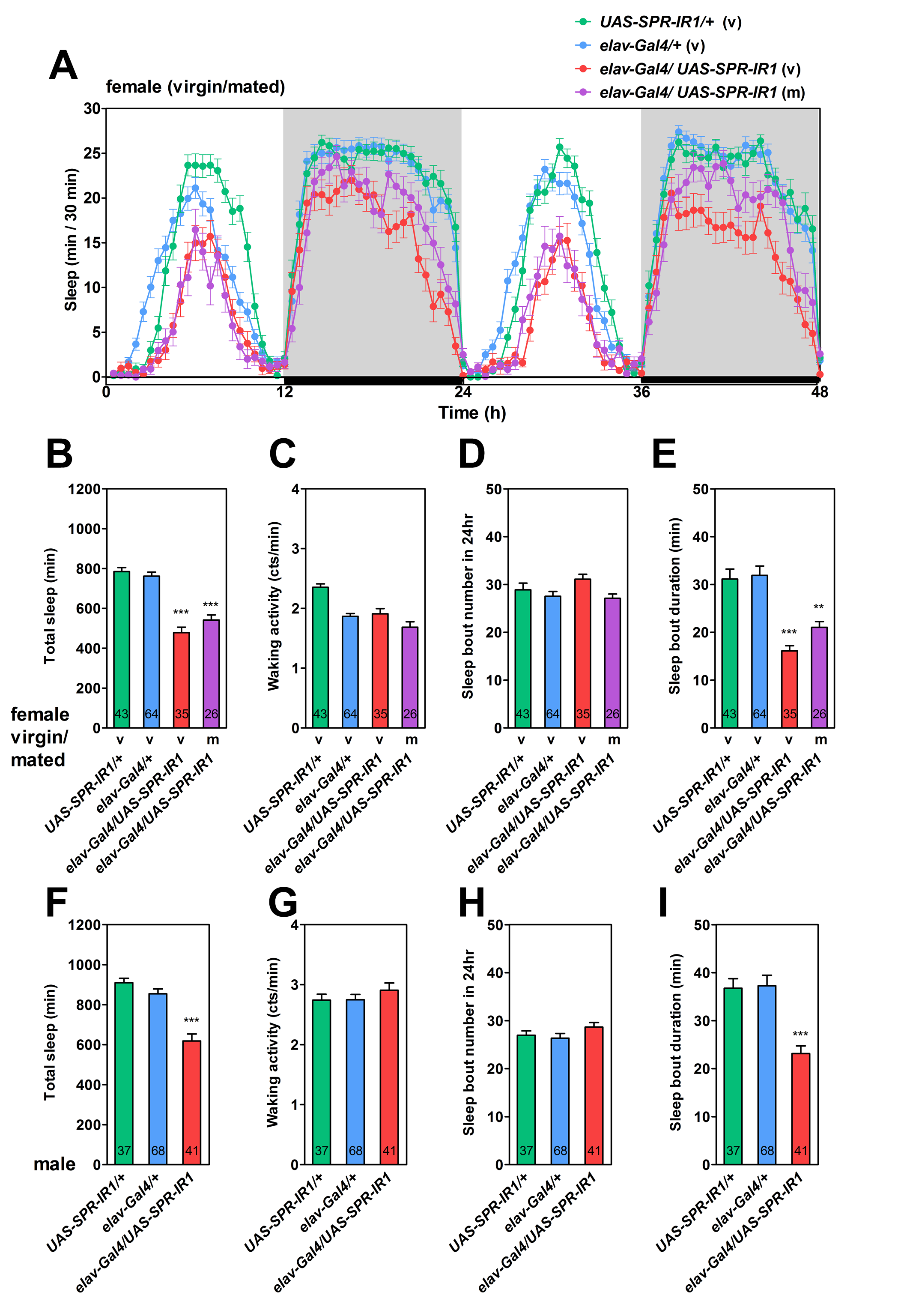

Supplement: Figure S3 — Like SPR deficient mutants, pan-neural SPR-RNAi flies also show defects in sleep maintenance (related to Figure 2 ). (A) Standard sleep plots of pan-neural SPR-RNAi (elav-Gal4, UAS-SPR-IR1) and its control females in a 12-h∶12-h light∶dark cycle (L∶D). Shaded boxes depict dark periods. (B–E) Sleep parameter of females of indicated genotypes. (F–I) Sleep parameter of males of indicated genotypes. (B, F) Total sleep duration per day. (C, G) Waking activity. (D, H) Sleep bout number per day. (E, I) Mean sleep-bout duration. Number in bars indicates n of the tested flies. Data are shown as means ± SEM. **, p<0.01; ***, p<0.001 for the comparison to both Gal4 and UAS controls by Student's t test (B–D, F–H) and Mann-Whitney U test (E, I). (TIF) [file pbio.1001974.s003.tif]

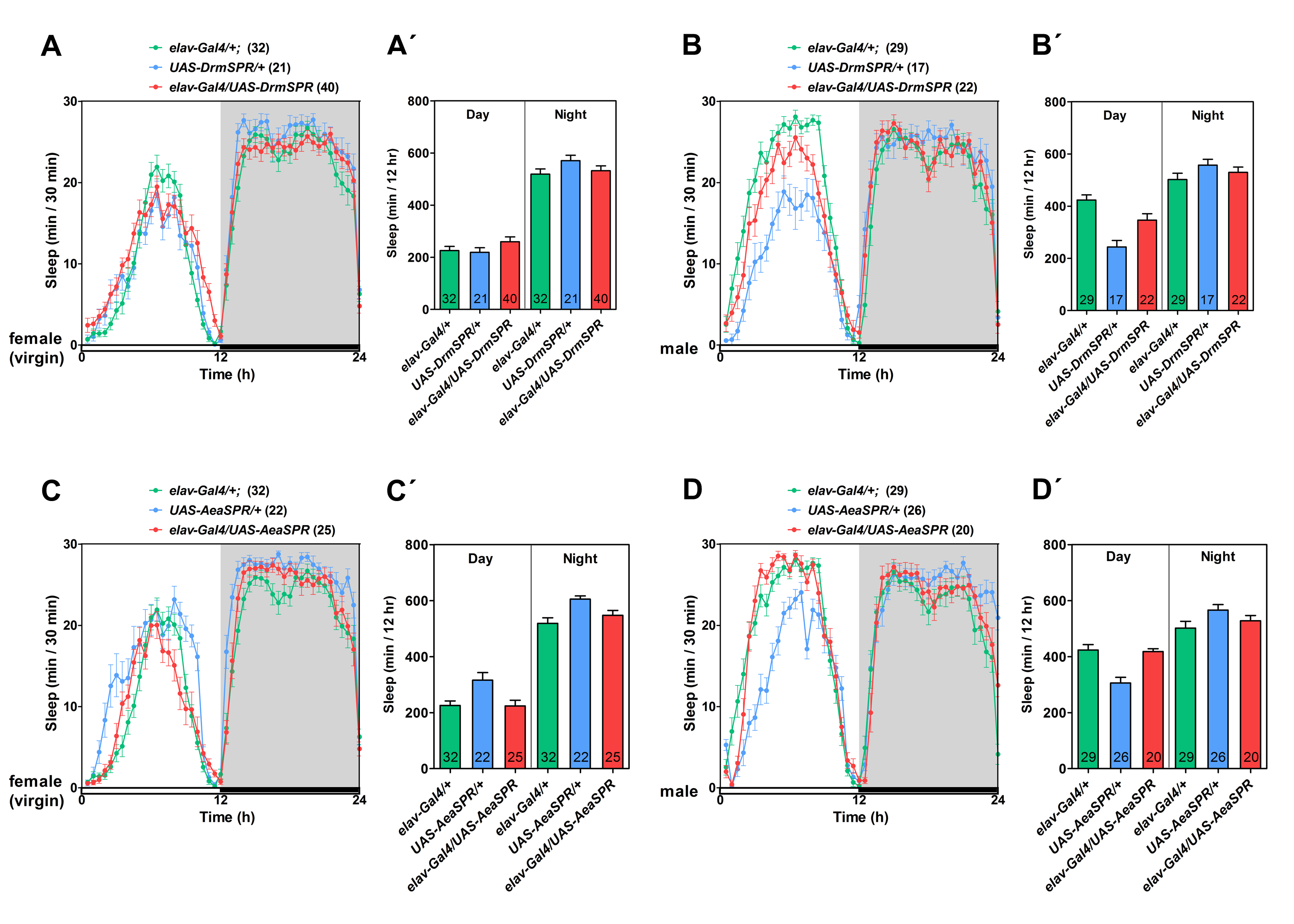

Supplement: Figure S4 — SPR overexpression alone in wild-type background does not elevate baseline sleep (related to Figure 2 ). (A–D) Standard sleep plots of virgin female (A, C) and males (B, D) of indicated genotypes. Shaded boxes depict dark periods. (A′–D′) Diurnal and nocturnal sleep durations of virgin females (A′, C′) and males (B′, D′) of indicated genotypes. Number in parentheses or bars indicates n of the tested flies. Data are shown as means ± SEM. All the comparisons to Gal4 and UAS controls are not significant (p>0.05, Student's t test). (TIF) [file pbio.1001974.s004.tif]

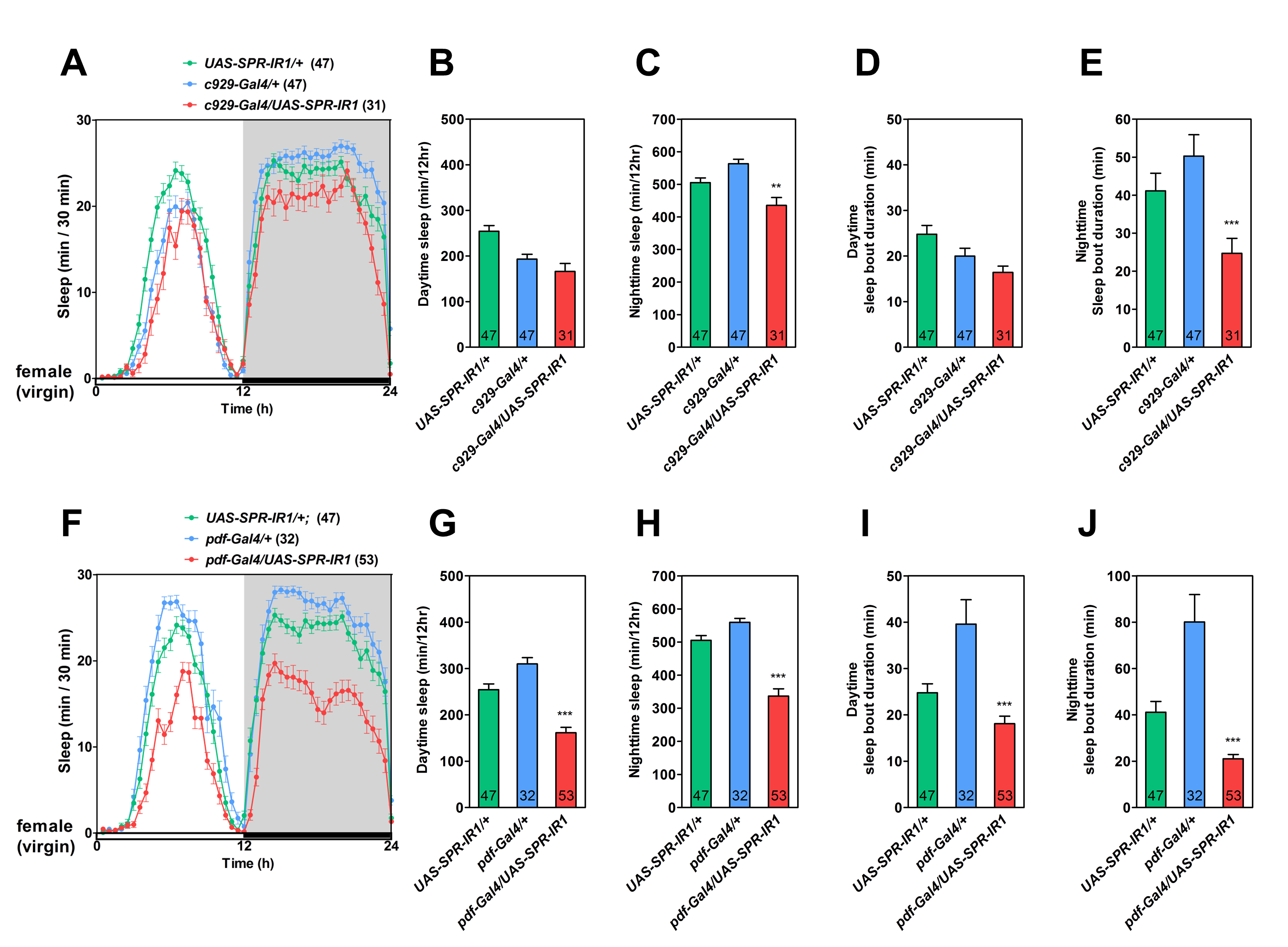

Supplement: Figure S5 — SPR expression in l-LNvs and s-LNvs is important for nocturnal and diurnal sleep, respectively (related to Figure 2 ). (A, F) Standard sleep plots of indicated genotypes of virgin females in a 12-h∶12-h light∶dark cycle (L∶D). Black bars in x-axis depict dark periods. (B, G) Daytime (ZT 0–12) sleep duration of indicated genotypes. (C, H) Night-time (ZT 12–24) sleep duration of indicated genotypes. (D, I) Average daytime (ZT 0–12) sleep-bout duration of indicated genotypes. (E, J) Average night-time (ZT 12–24) sleep-bout duration of indicated genotypes. Number in parentheses or bars indicates n of the tested flies. Data are shown as means ± SEM. **, p<0.01; ***, p<0.001 for the comparison to both Gal4 and UAS controls by Student's t test (B–C, G–H) and Mann-Whitney U test (D–E, I–J). Dataset used for Figure 2A is reanalysed. (TIF) [file pbio.1001974.s005.tif]

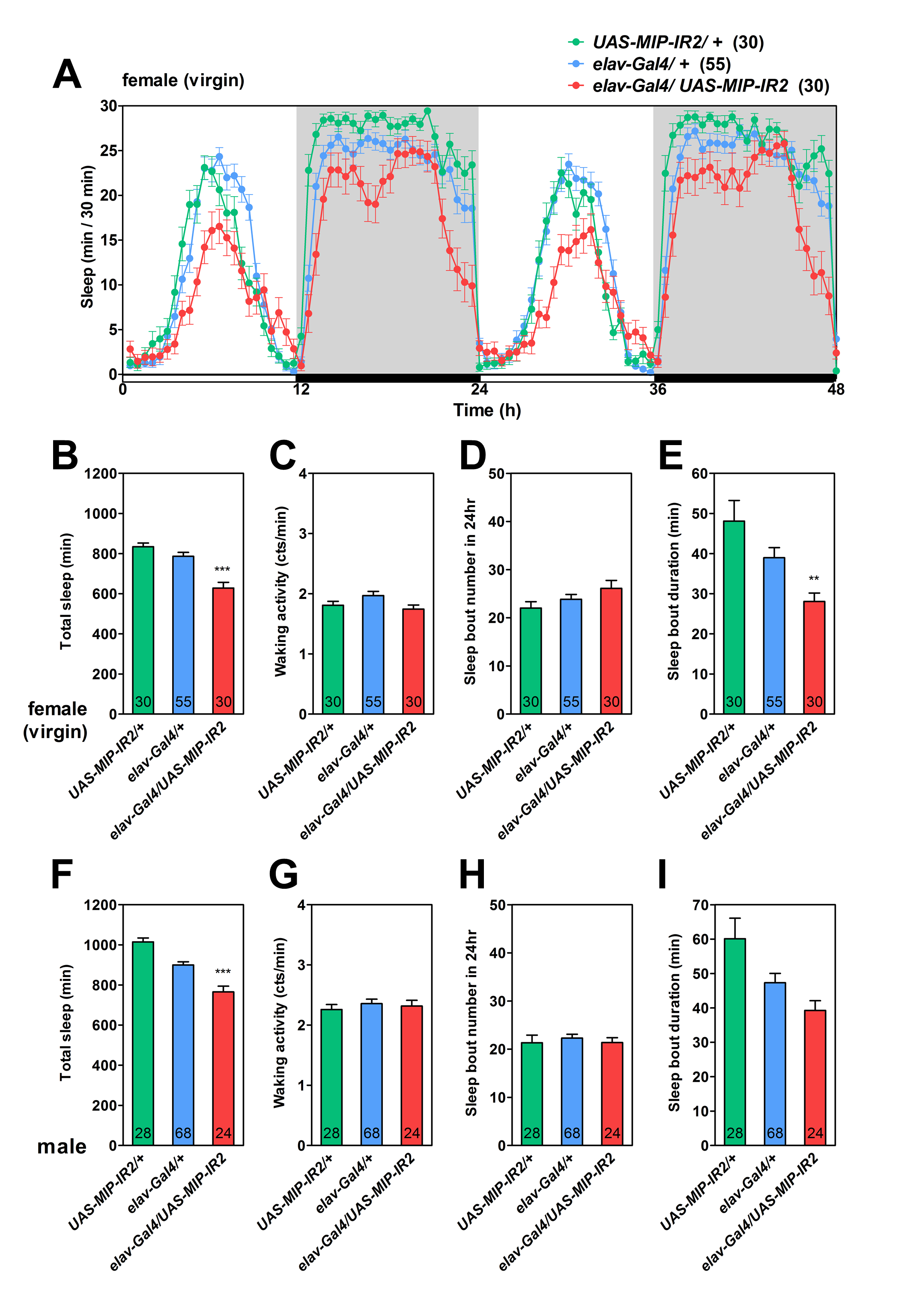

Supplement: Figure S6 — Like UAS-MIP-IR1 , UAS-MIP-IR2 combined with elav-Gal4 also shortens total sleep by impairing sleep maintenance (related to Figure 3 ). (A) Standard sleep plots of pan-neural MIP-RNAi (elav-Gal4, UAS-MIP-IR2) and its control females in a 12-h∶12-h light∶dark cycle (L∶D). Shaded boxes depict dark periods. (B–E) Sleep parameter of females of indicated genotypes. (F–I) Sleep parameter of males of indicated genotypes. (B, F) Total sleep duration per day. (C, G) Waking activity. (D, H) Sleep bout number per day. (E, I) Mean sleep-bout duration. Number in parentheses or bars indicates n of the tested flies. Data are shown as means ± SEM. **, p<0.01; ***, p<0.001 for the comparison to both Gal4 and UAS controls by Student's t test (B–D, F–H) and Mann-Whitney U test (E, I). (TIF) [file pbio.1001974.s006.tif]

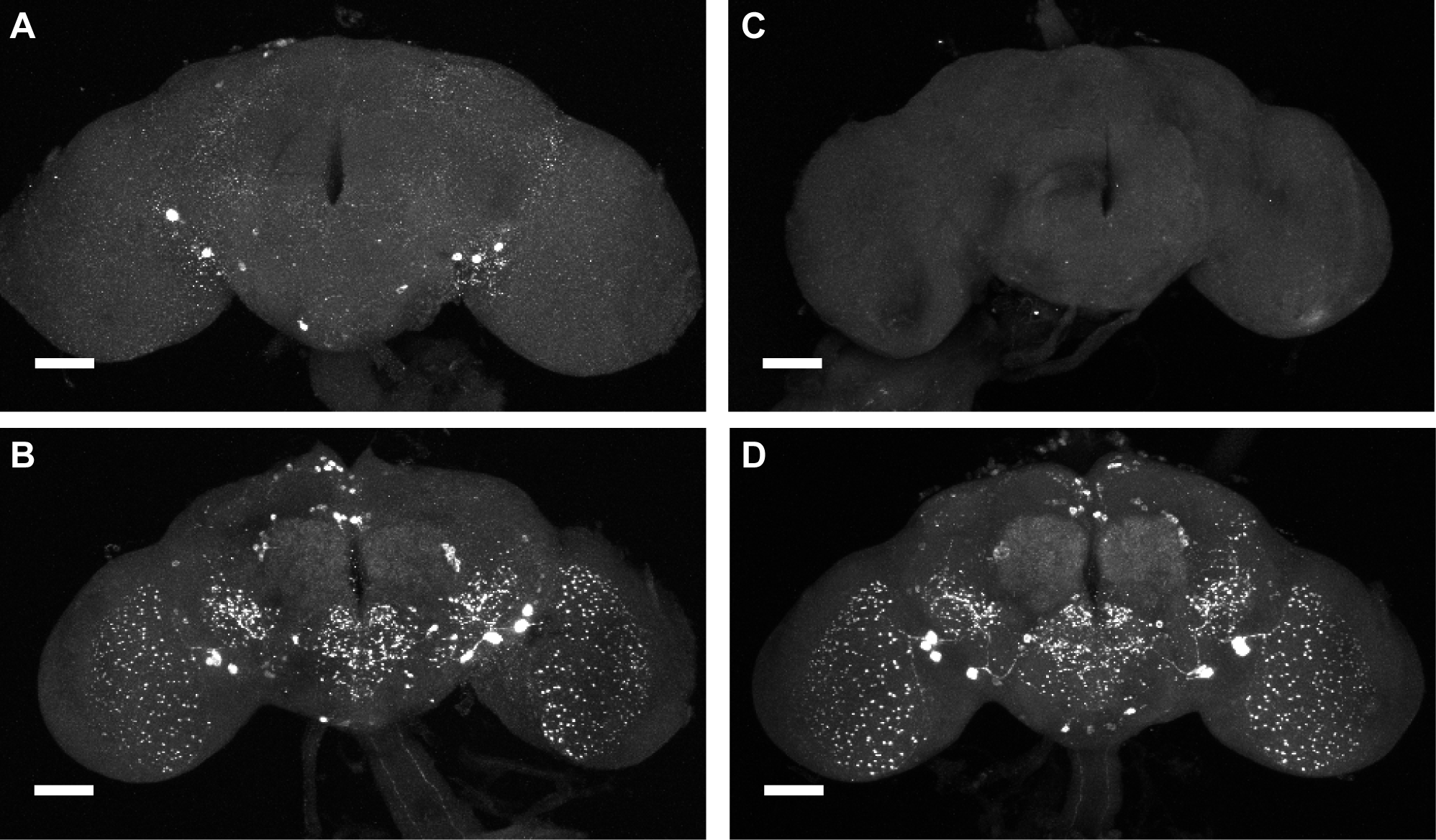

Supplement: Figure S7 — Anti-MIP staining is greatly attenuated in two MIP-RNAi lines (related to Figures 3 and S6). The brain anti-MIP staining of elav-Gal4 UAS-MIP-IR1 (A), UAS-MIP-IR1 (B), elav-Gal4 UAS-MIP-IR2 (C), and UAS-MIP-IR2 (D). Scale bars, 50 µm. (TIF) [file pbio.1001974.s007.tif]

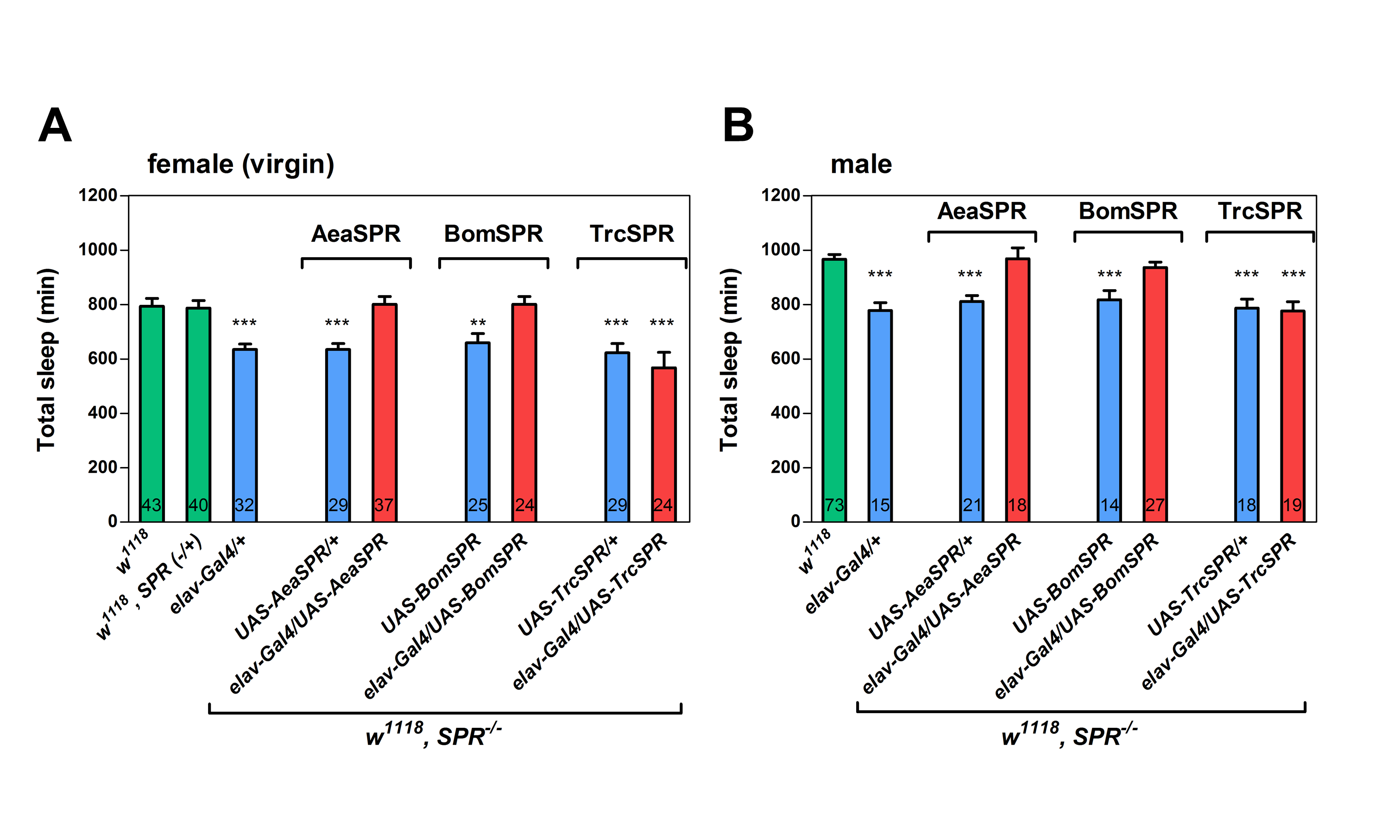

Supplement: Figure S8 — Drosophila SPR can be replaced with insect SPRs less sensitive to SP (related to Figure 4 ). (A, B) Total sleep duration per day of virgin females (A) and males (B) of indicated genotypes. AeaSPR, BomSPR, and TrcSPR indicate SPRs from a mosquito A. aegypti, a moth B. mori, and a beetle Tribolium castaneum, respectively. Note that AeaSPR and BomSPR have strong sensitivity toward MIP, but intermediate or low sensitivity toward SP (for details, see text). TrcSPR, insensitive to either MIP or SP is used as a control. Number in parentheses indicates n of the tested flies. Data are shown as means ± SEM. **, p<0.01; ***, p<0.001 for the comparison to w1118 control by Student's t test. (TIF) [file pbio.1001974.s008.tif]

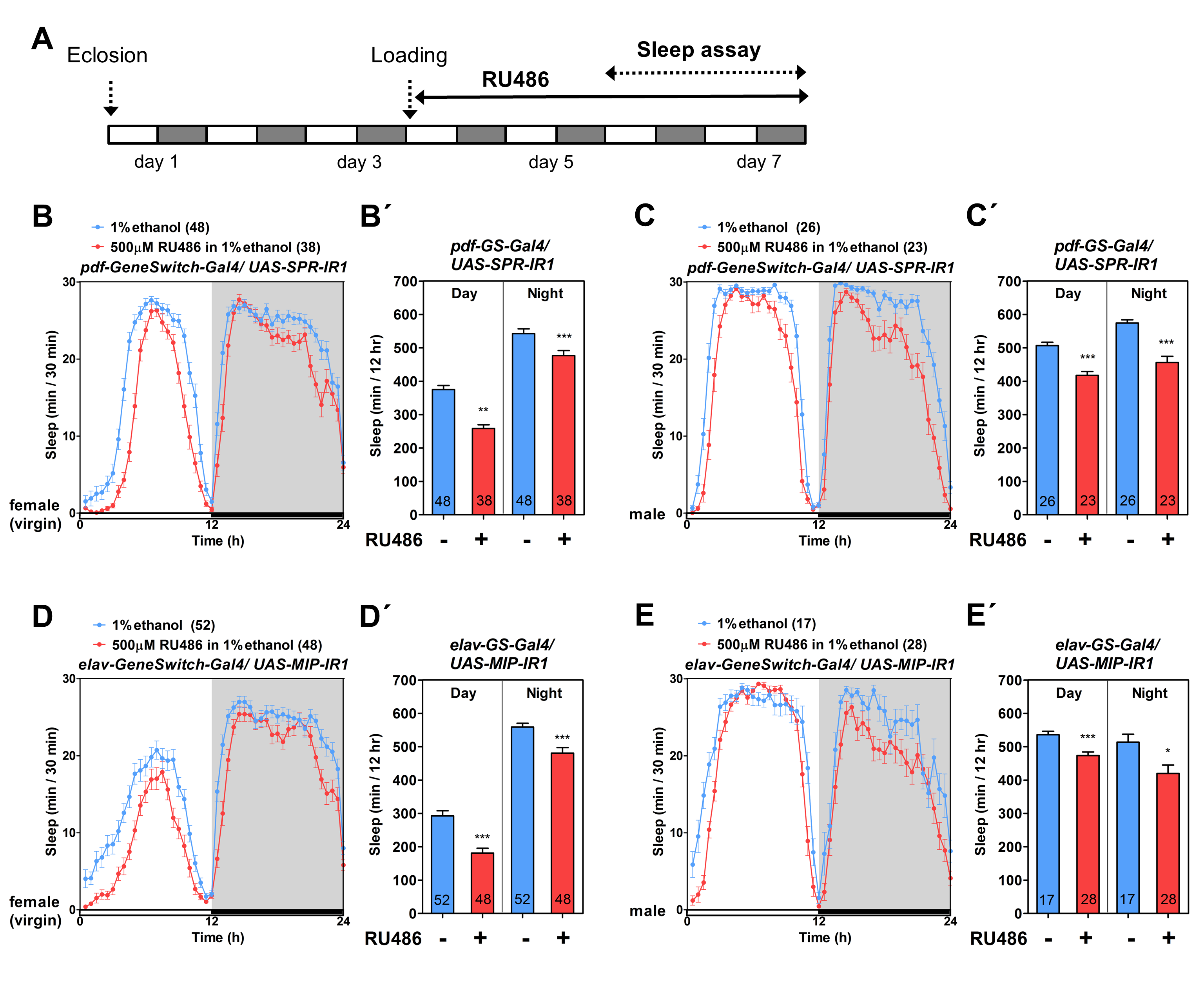

Supplement: Figure S9 — Adult-specific knockdown of SPR or MIP reduces diurnal and nocturnal sleep in both sexes (related to Figures 1 and 3 ). (A) Protocol for behavioral experiments in (B–E). RU486 treatment activates Gal4 expression in flies carrying GeneSwitch-Gal4. (B–E) Standard sleep plots of virgin females (B, D) and males (C, E) of indicated genotypes. (B′–E′) Diurnal and nocturnal sleep durations of virgin females (B′, D′) and males (C′, E′) of indicated genotypes. ‘+’ and ‘−’ indicate RU486 and vehicle treatment, respectively. Numbers in parentheses or bars indicate n of the tested flies. Data are shown as means ± SEM. *, p<0.05; **, p<0.01; ***, p<0.001 for the comparison between RU486 and vehicle by Student's t test. (TIF) [file pbio.1001974.s009.tif]

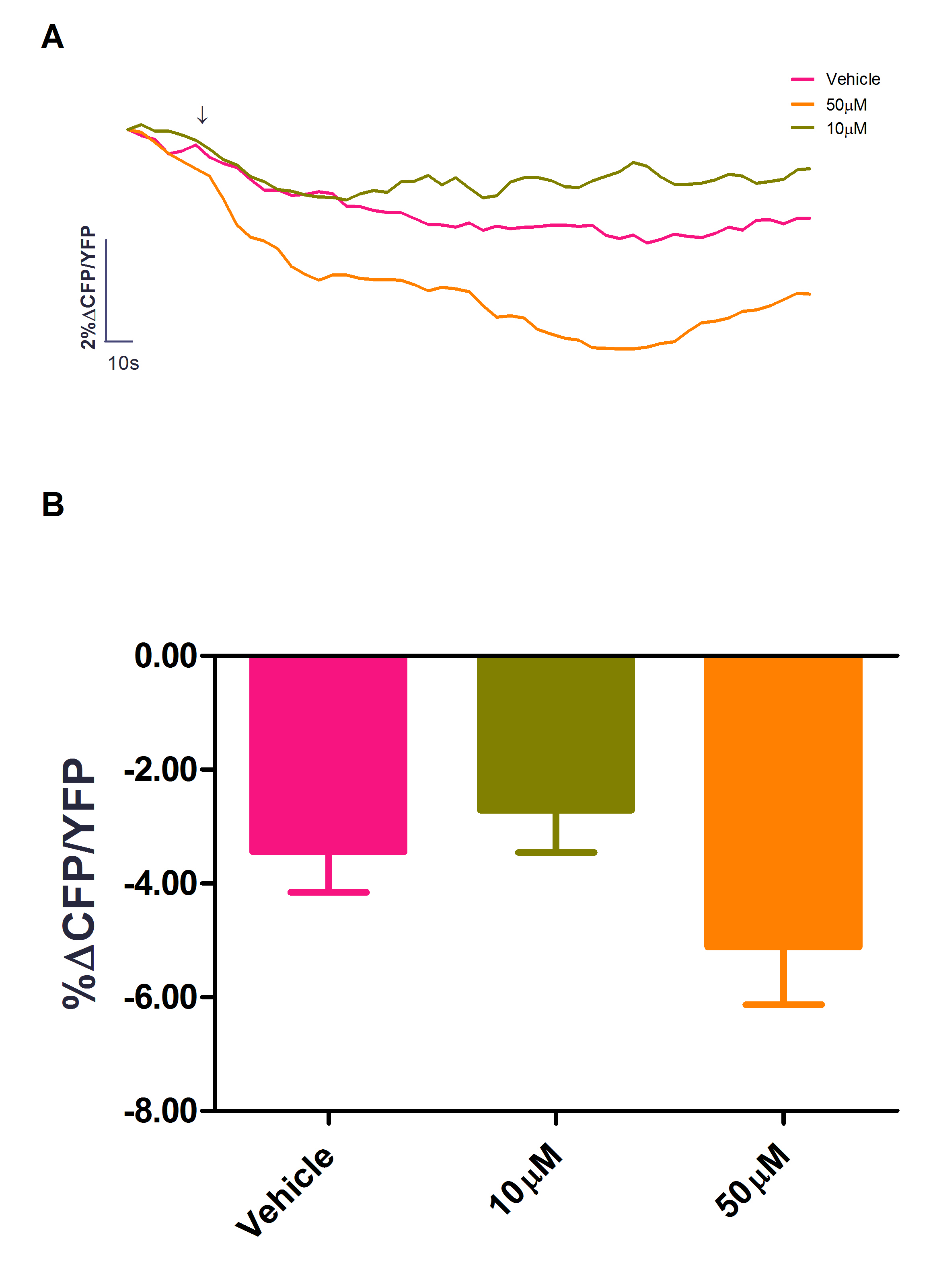

Supplement: Figure S10 — The effects of MIP on cAMP dynamics within the s-LNvs. (A) Averaged Epac1-camps YFP/CFP FRET plots of s-LNvs from pdf-Gal4,UAS-Epac1-camps flies in response to 10 and 50 µM MIP doses applied as indicated by the arrow. (B) A summary of the average maximum loss of Epac-1-camps CFP/YFP for the data shown in (A) between 30 and 120 s. A one-way ANOVA revealed no significant effect of MIP concentration for the s-LNvs (p<0.1290) on maximum loss of CFP/YFP ratio. A Dunn's multiple comparison test revealed no significant differences (p>0.05) between vehicle controls and the 10, 50 µM MIP treatments. The sample sizes for (A) and (B) were as follows: for vehicle, ten neurons from nine brains (10, 9), 10 µM MIP (13, 8), 50 µM MIP (14, 11). (TIF) [file pbio.1001974.s010.tif]

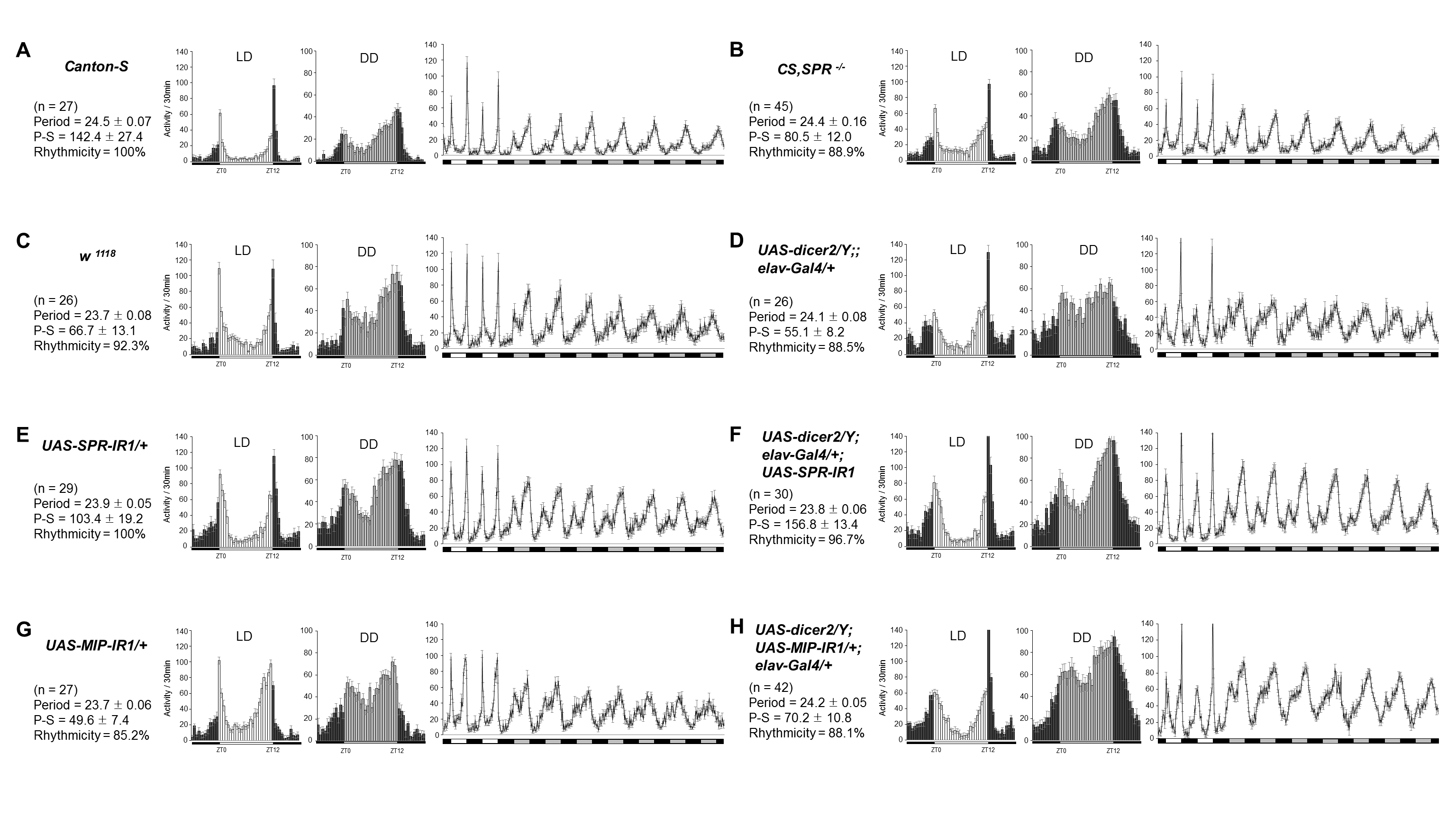

Supplement: Figure S11 — Circadian activities of flies lacking either SPR or MIP are normal. Average activity profiles in LD (left), and DD (middle) conditions, and average actograms throughout the behavioral analysis (right) of indicated genotypes. Note none of tested lines show obvious defects in circadian rhythms, morning and evening anticipations both LD and DD condition. Alternating white and black bar in the x-axis indicates LD cycle (12-h∶12-h), whereas gray and black bars indicate DD cycle. (TIF) [file pbio.1001974.s011.tif]

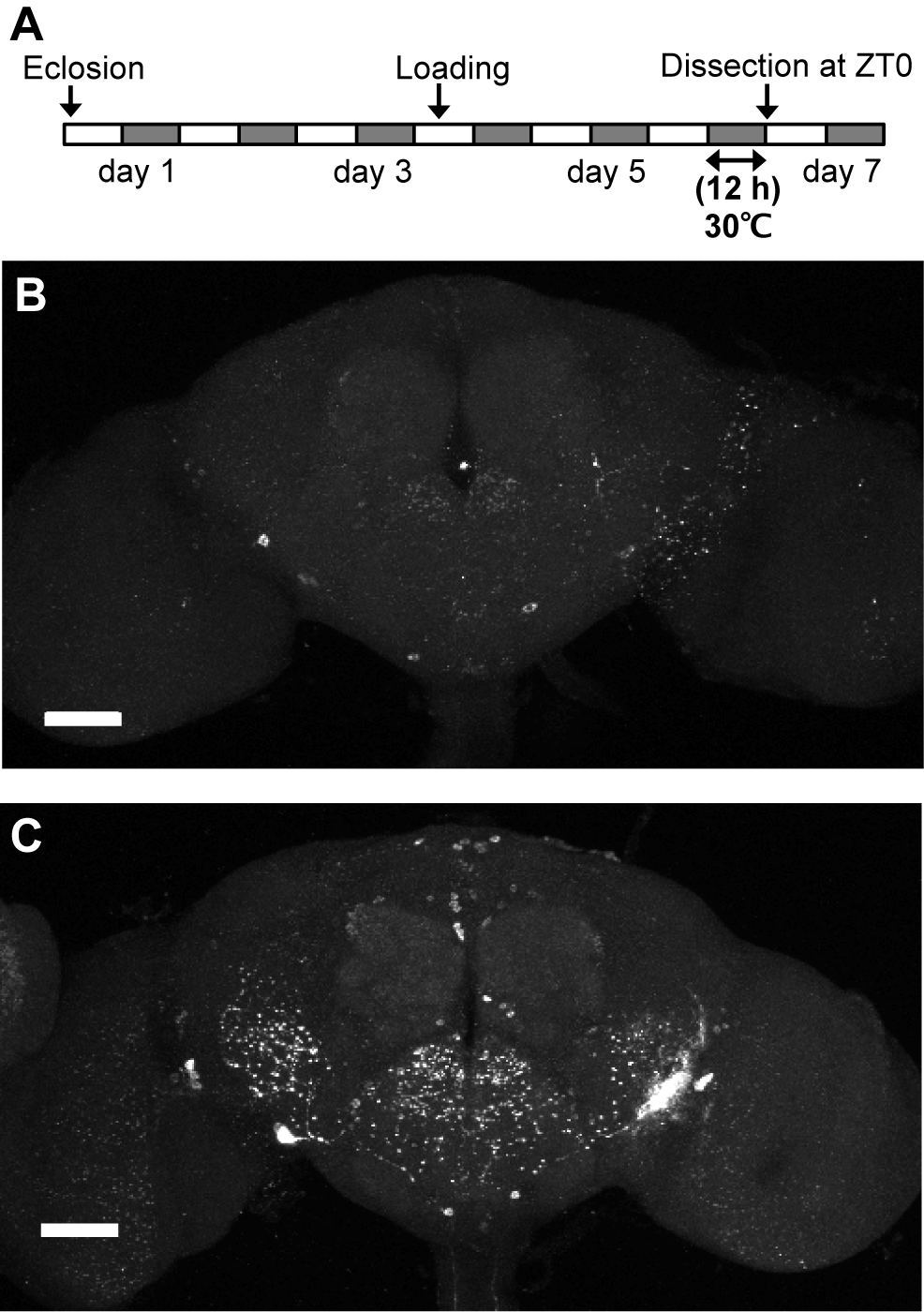

Supplement: Figure S12 — Activation of MIP neurons results in complete depletion of anti-MIP labeling in the brain (Related to Figure 5 ). (A) The experimental protocol. Five-day-old males were subjected to thermo-activation at 30°C from ZT 12 to ZT 24, and their CNS were dissected and processed shortly thereafter. (B, C) The anti-MIP stained brain of MIP-Gal4 UAS-dTrpA1 (B) or UAS-dTrpA1 control males (C) subjected to the thermal activation. Scale bars, 50 µm. (TIF) [file pbio.1001974.s012.tif]

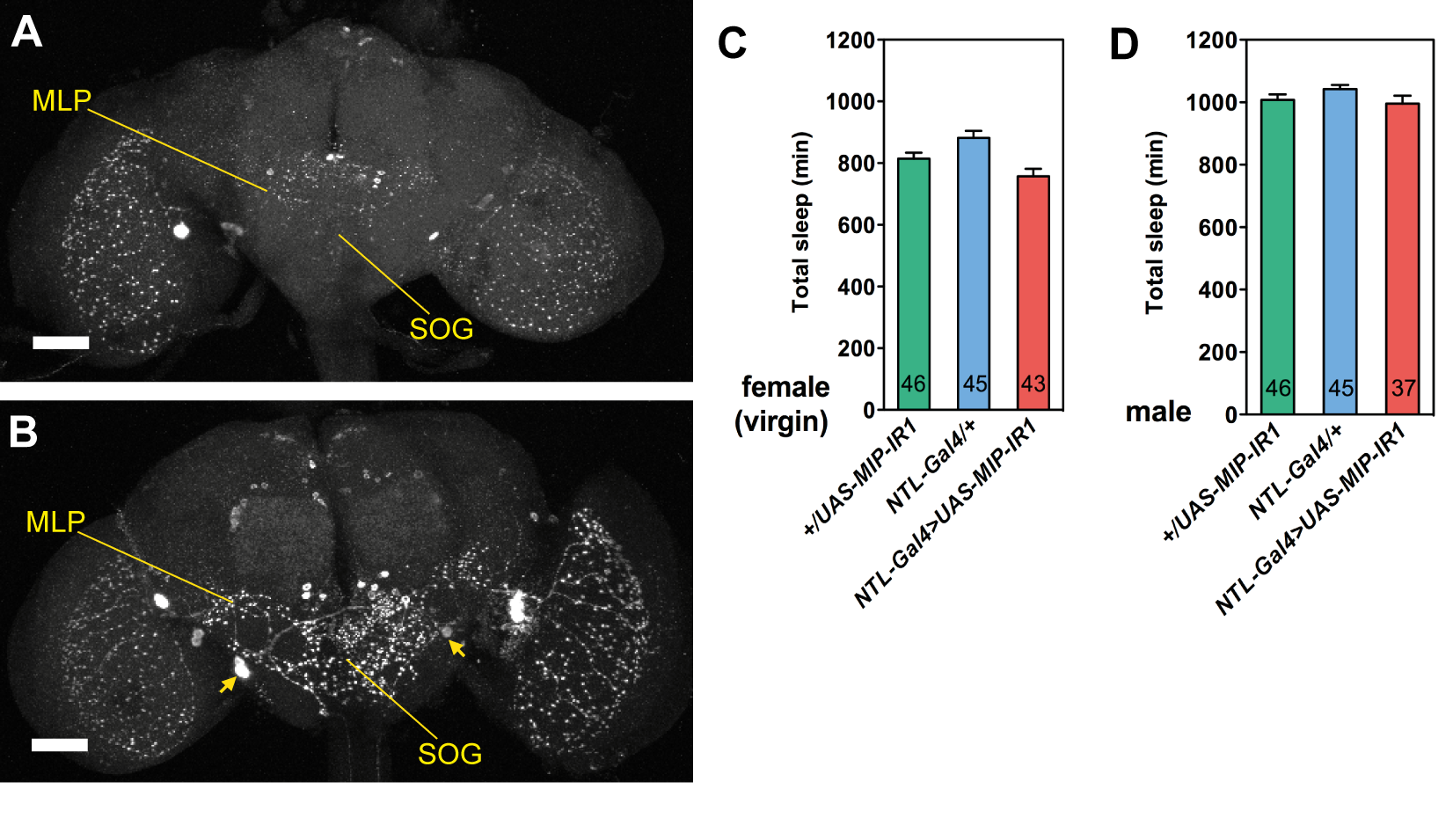

Supplement: Figure S13 — MIP expression in MIP-ICLI is not important for sleep regulation (related to Figure 6 ). (A, B) The brain of NTL-Gal4 UAS-MIP-IR1 (A) or NTL-Gal4 control males (B) stained with anti-MIP. Note MIP expression in the MLP and SOG is greatly attenuated in the MIP-RNAi targeted by NTL-Gal4, confirming MIP-ICLI neurons (arrows) innervating the MLP and SOG express NTL-Gal4. (C, D) Total daily sleep duration of females (C) and males (D) of indicated genotypes. Number in bars indicates n of the tested flies. Data are shown as means ± SEM. Not significant (p>0.05) for the comparison to both Gal4 and UAS controls by one-way ANOVA with Tukey's post hoc test. Scale bars, 50 µm. (TIF) [file pbio.1001974.s013.tif]
