# Supplementary material for: A Homeostatic Sleep-Stabilizing Pathway in Drosophila Composed of the Sex Peptide Receptor and Its Ligand, the Myoinhibitory Peptide
Source: PLoS Biol. 2014 Oct 21;12(10):e1001974. doi: 10.1371/journal.pbio.1001974 (PMC4204809; doi:10.1371/journal.pbio.1001974)
Supplement: Table S1 — Circadian rhythm parameters of SPR and MIP mutants and RNAi. (DOCX) [file pbio.1001974.s014.docx]

Table S1. Circadian rhythm parameters of SPR and MIP mutants and RNAi

| Genotypes | *n ^b^* | AR (*n)* | R (*n)* | Rhythmicity ^c^ | Tau | SEM | p-s ^d^ | SEM |
| --- | --- | --- | --- | --- | --- | --- | --- | --- |
| *CS* | 27 | 0 | 27 | 100% | 24.5 | 0.07 | 142.4 | 27.4 |
| *CS, SPR^-/-^* | 45 | 5 | 40 | 88.9% | 24.4 | 0.16 | 80.5 | 12.0 |
| *w^1118^* | 26 | 2 | 24 | 92.3% | 23.7 | 0.08 | 66.7 | 13.1 |
| *UAS-dicer2/Y;; elav-Gal4/+* | 26 | 3 | 23 | 88.5% | 24.1 | 0.08 | 55.1 | 8.2 |
| *UAS-SPR-IR1/ +* | 29 | 0 | 29 | 100% | 23.9 | 0.05 | 103.4 | 19.2 |
| *UAS-dicer2/Y; elav-Gal4/ +; UAS-SPR-IR1/ +* | 30 | 1 | 29 | 96.7% | 23.8 | 0.06 | 156.8 | 13.4 |
| *UAS-MIP-IR1/ +* | 27 | 4 | 23 | 85.2% | 23.7 | 0.06 | 49.6 | 7.4 |
| *UAS-dicer2/Y; UAS-MIP-IR1/+; elav-GAL4/+* | 42 | 5 | 37 | 88.1% | 24.2 | 0.05 | 70.2 | 10.8 |

^a^ Flies were kept at 25°C and exposed to 3 day of 12 h:12 h LD followed by 10 day of DD.

^b^ Total number of flies that survived until the end of the test.

^c^ Percentage of flies with activity rhythms having a P-S value of > 10.

^d^ (P-S) Power - Significant.
