# Supplementary material for: A Homeostatic Sleep-Stabilizing Pathway in Drosophila Composed of the Sex Peptide Receptor and Its Ligand, the Myoinhibitory Peptide
Source: PLoS Biol. 2014 Oct 21;12(10):e1001974. doi: 10.1371/journal.pbio.1001974 (PMC4204809; doi:10.1371/journal.pbio.1001974)
Supplement: Text S1 — Supplemental materials and methods. (DOCX) [file pbio.1001974.s015.docx]

**Supplemental Materials and Methods**

**Circadian assay**

To For circadian behavioural assay, 30 min-bin locomotor activity was measured with DAM System monitors (Trikinetics). After prior entrainment in 12 h light:dark (LD) cycle, flies were monitored for 2~5 days under LD cycle and followed constant dark (DD) cycles for a week. Data were analysed with Clocklab analysis software (Actimetrics) as described previously [1].

**Conditional neuronal activation using *GeneSwitch-Gal4* lines**

For induction of *elav-GeneSwitch-Gal4* [2] and *pdf-GeneSwitch-Gal4* drivers [3], flies were fed 500 µM RU486 (mifepristone, Sigma, St. Louis, MO. Catalog number: H110-01) in 1% EtOH for 2 days in 4% sucrose and 2% agarose tubes. As a control, we fed 1% EtOH (with 4% sucrose/ 2% agarose) for 2 days in a same chamber.

**Supplemental References**

1. Lim C, Chung BY, Pitman JL, McGill JJ, Pradhan S, et al. (2007) Clockwork orange encodes a transcriptional repressor important for circadian-clock amplitude in *Drosophila*. Curr Biol 17: 1082–1089.

2. Osterwalder T, Yoon KS, White BH, Keshishian H (2001) A conditional tissue-specific transgene expression system using inducible GAL4. Proc Natl Acad Sci USA 98: 12596–12601.

3. Depetris-Chauvin A, Berni J, Aranovich EJ, Muraro NI, Beckwith EJ, et al. (2011) Adult-specific electrical silencing of pacemaker neurons uncouples molecular clock from circadian outputs. Curr Biol 21: 1783–1793.
